# FASCICLIN-LIKE 18 Is a New Player Regulating Root Elongation in *Arabidopsis thaliana*

**DOI:** 10.3389/fpls.2021.645286

**Published:** 2021-04-07

**Authors:** Hewot Allelign Ashagre, David Zaltzman, Anat Idan-Molakandov, Hila Romano, Oren Tzfadia, Smadar Harpaz-Saad

**Affiliations:** ^1^The Robert H. Smith Institute of Plant Sciences and Genetics in Agriculture, The Hebrew University of Jerusalem, Jerusalem, Israel; ^2^Faculty of Pharmaceutical, Biomedical and Veterinary Sciences, Institute for Tropical Medicine, Antwerp, Belgium

**Keywords:** fasciclin-like, arabinogalactan protein, cell wall, root elongation, ABA, abiotic stress

## Abstract

The plasticity of root development represents a key trait that enables plants to adapt to diverse environmental cues. The pattern of cell wall deposition, alongside other parameters, affects the extent, and direction of root growth. In this study, we report that FASCICLIN-LIKE ARABINOGALACTAN PROTEIN 18 (FLA18) plays a role during root elongation in *Arabidopsis thaliana*. Using root-specific co-expression analysis, we identified *FLA18* to be co-expressed with a sub-set of genes required for root elongation. *FLA18* encodes for a putative extra-cellular arabinogalactan protein from the *FLA*-gene family. Two independent T-DNA insertion lines, named *fla18-1* and *fla18-2*, display short and swollen lateral roots (LRs) when grown on sensitizing condition of high-sucrose containing medium. Unlike *fla4/salt overly sensitive 5* (*sos5*), previously shown to display short and swollen primary root (PR) and LRs under these conditions, the PR of the *fla18* mutants is slightly longer compared to the wild-type. Overexpression of the *FLA18* CDS complemented the *fla18* root phenotype. Genetic interaction between either of the *fla18* alleles and *sos5* reveals a more severe perturbation of anisotropic growth in both PR and LRs, as compared to the single mutants and the wild-type under restrictive conditions of high sucrose or high-salt containing medium. Additionally, under salt-stress conditions, *fla18sos5* had a small, chlorotic shoot phenotype, that was not observed in any of the single mutants or the wild type. As previously shown for *sos5*, the *fla18-1* and *fla18-1sos5* root-elongation phenotype is suppressed by abscisic acid (ABA) and display hypersensitivity to the ABA synthesis inhibitor, Fluridon. Last, similar to other cell wall mutants, *fla18* root elongation is hypersensitive to the cellulose synthase inhibitor, Isoxaben. Altogether, the presented data assign a new role for FLA18 in the regulation of root elongation. Future studies of the unique vs. redundant roles of FLA proteins during root elongation is anticipated to shed a new light on the regulation of root architecture during plant adaptation to different growth conditions.

## Introduction

Developmental pliancy allows plants to respond to a wide range of environmental signals by altering the pattern of plant growth and development. This enables otherwise immobile plants to adjust to heterogeneous soil and ever-changing environmental conditions by continuous forage toward optimal conditions with respect to water, nutrients, and other resources. In practice, this is achieved by modification of root system architecture in response to different developmental and environmental cues. The adaptability and flexibility of the root system is one of the main determinants affecting the rate and extent of plant biomass production (Lynch, 1995; Malamy, 2005; Gruber et al., 2013; Tian et al., 2014; Slovak et al., 2016).

The plasticity of root development is obtained through embryonic and postembryonic organogenesis. The primary root (PR) apical meristem, which is responsible for PR growth, is formed as part of the developing embryo. In contrast, lateral roots (LRs) are repeatedly formed from the PR, or from previously formed LRs, and determine the shape of the plant root system dictating the efficiency of plant anchorage, water uptake and nutrient acquisition. It is noteworthy, that first-order LRs are formed from the PR, while second- and third-order LRs are formed from lower-order LRs. LR meristems are formed post-embryonically from the pericycle, a highly specified cell layer encircling the root vascular stele tissue. The intervals of LR formation vary according to developmental stage and growth conditions (Malamy and Benfey, 1997; Casimiro et al., 2001; De Smet et al., 2003; Lavenus et al., 2013; Vilches-Barro and Maizel, 2015; Slovak et al., 2016). While much is known about the mechanism employed in LR initiation and meristem formation, little is known about the mechanisms employed in PR vs. LR elongation and how it is modified in response to different environmental signals.

Cell expansion can occur through isotropic growth in which the cell expands to the same extent in all directions, or through anisotropic growth in which the cell expends in a non-uniform, directional manner. Root elongation is obtained through anisotropic cell expansion. While turgor pressure provides the driving force for this process, the pattern of cell wall deposition in the extra-cellular matrix is one of the parameters dictating growth extent and direction. The current model suggests that anisotropic cell expansion is obtained as a result of stiffer cell wall deposited perpendicular to the growth axis, yielding cell elongation driven by the cytosolic turgor pressure (Green, 1980; Taiz, 1984; Darley et al., 2001; Baskin, 2005). Primary cell wall is deposited in all plant cells and protect the protoplast as the cell grows, while secondary cell wall is synthesized in specific cell types as part of the process of cell differentiation which takes place after the cell reached its final size. The plant cell walls are composed primarily of three classes of polysaccharides: cellulose, hemicelluloses and pectins (Bacic et al., 1988; Carpita and Gibeaut, 1993; Cosgrove, 2005; Somerville, 2006). It was previously shown that cellulose synthesis, organization and cross-linking with other cell wall material is a key determinant dictating the extent of anisotropic cell expansion and subsequently, of root elongation (Arioli et al., 1998; Fagard et al., 2000; Schindelman et al., 2001). Cellulose is composed of β-(1,4)-linked glucan chains synthesized at the plasma membrane by the cellulose synthase complex (CSC; Mueller et al., 1976; Brown et al., 1996; Kimura et al., 1999). Each glucan chain is synthesized by CELLULOSE SYNTHASE A (CESA), a glycosyltransferase encoded by multi gene-families in different plant species (Pear et al., 1996; Delmer, 1999; Richmond and Somerville, 2000; McFarlane et al., 2014). In *Arabidopsis*, *CESA1*, *CESA3*, and *CESA6* have been shown to be essential for cellulose synthesis as part of primary cell wall deposition during root elongation. Mutants in either of these three *CESAs*, display short roots with a swollen root tip (Hauser et al., 1995; Caño-Delgado et al., 2000; Fagard et al., 2000; Desprez et al., 2007). Similar results were obtained using chemical inhibitors of CESA (Heim et al., 1989; Desprez et al., 2002; Tateno et al., 2016). Additional components required for cell wall deposition and organization have been shown to play a role during root elongation, including KORRIGAN (KOR), COBRA (COB), FASCICLIN-LIKE 4/SALT-OVERLY SENSITIVE 5 (FLA4/SOS5), CHITINASE-LIKE 1 (CTL1), and others (Nicol, 1998; Schindelman et al., 2001; Shi et al., 2003; Hermans et al., 2010). However, so far the intricate mechanism regulating cell wall synthesis and remodeling in elongating roots under different growth conditions has not been fully established.

To identify new candidate-genes participating in cell wall deposition during root elongation, a tissue specific co-expression analysis was performed as part of the current study. The premise of this method is the well established phenomenon that genes functioning in the same pathway, or required for the same process, tend to express in a transcriptionally coordinated manner. Co-expression approaches have been used to assign function for genes involved in cellulose synthesis, lignin deposition, and other metabolic processes (Brown et al., 2005; Ruprecht et al., 2011; Yang et al., 2011, 2019; Wang et al., 2012; Ben-Tov et al., 2015; Endler et al., 2015; Ransbotyn et al., 2015; Voiniciuc et al., 2015a, b, 2018). In this work, ten genes, known to play a role in primary cell wall deposition during root elongation, were used as ‘baits’ to mine a spatio-temporal, high-resolution root gene expression dataset (Supplementary Table 1; Brady et al., 2007). The identified candidate genes were examined for perturbation of root elongation. Interestingly, two independent T-DNA insertion lines corresponding to different mutations in the *FASCICLIN-LIKE ARABINOGALACTAN 18* (*FLA18*) gene (AT3G11700), which has not been assigned a function so far, showed modification of PR and LR elongation under different growth conditions. The presented data suggest that FLA18 plays a role in the regulation of root elongation.

## Results

### Tissue-Specific Co-expression Analysis Suggests That *FLA18* May Play a Role in Root Elongation

A tissue specific co-expression analysis was performed using the *Arabidopsis* high-resolution spatio-temporal gene expression dataset generated for developing root (Brady et al., 2007). The ‘bait’ list for the co-expression analysis was generated based on genes known in the literature to be involved in primary cell wall deposition during *root* elongation (Supplementary Table 1). Pearson correlation identifies a tight correlation between *FLA18* gene expression and that of *CESA1*, *CESA3*, and *CESA6* involved in primary cell wall deposition during root elongation (Supplementary Figure 2B; Fagard et al., 2000; Desprez et al., 2007; Persson et al., 2007; Chen et al., 2016). A highly specific expression pattern was observed for representative ‘baits’ and *FLA18* along the longitudinal root axis (Supplementary Figure 2A). This includes, low expression at the meristematic zone, high expression at the transition from the meristematic to the elongation zone and then again, reduced expression in the interface between the elongation and the differentiation zone (Supplementary Figure 2A). In contrast, *CESA4*, *CESA7* or *CESA8*, involved in secondary cell wall deposition as part of root vascular system differentiation, display a very different expression pattern (Supplementary Figure 2A). No correlation in gene expression was observed between *FLA18* and *CESA4*, *CESA7*, or *CESA8* using the root-specific data-set (Supplementary Figure 2B; Turner and Somerville, 1997; Persson et al., 2005; Taylor-Teeples et al., 2015; Meents et al., 2018). In addition, the expression of *FLA18* and other ‘baits’ used for the co-expression analysis (*CESA1, CESA3*, and *CESA6* are presented as an example) is induced in the linage of cells destined to differentiate into root hairs, and to a lower extent in cell layers with non-root hair identity (Supplementary Figure 2A). Taken together, the co-expression relationship between *FLA18* and established-players required for root elongation prompted us to further investigate the role of *FLA18* in this developmental context.

### *FLA18* Gene Expression

To study *FLA18* expression pattern throughout plant growth and development, we used Reverse Transcription PCR (RT-PCR) analysis and a GUS-reporter gene driven by the *FLA18* promoter. The *FLA18* transcript was detected in all organs, including leaves, stems, siliques, and flowers (Supplementary Figures 3A,B). GUS activity was detected in various developmental contexts, correlating with the RT-PCR results (Supplementary Figures 3C–F). The GUS staining identified *FLA18* expression in stems, flowers, as well as elongating tissues like, root tips, and hypocotyls of dark-grown seedlings. In dark-grown seedlings, the induction of *FLA18* gene expression was detected specifically in the upper part of the hypocotyl, which was previously shown to display high rate of cell elongation as compared to other parts of the hypocotyl (Crowell et al., 2011). Overall, *FLA18* gene expression was confirmed in elongating roots and additional developmental contexts throughout plant development.

### *fla18* Mutants Display Conditional Perturbation of Lateral Root Elongation

**FIGURE 1:**
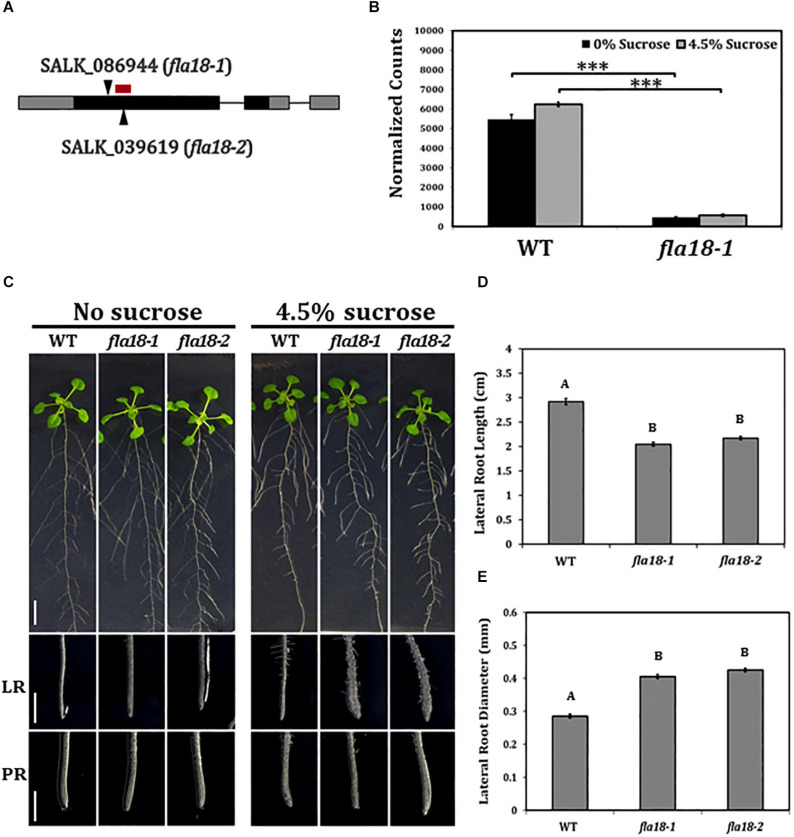
*fla18* mutants display conditional perturbation of root elongation. **(A)** Schematic presentation of the *FLA18* gene as annotated by The Arabidopsis Information Resource, pinpointing the location of the T-DNA insertion in both alleles examined, i.e., *fla18-1* (SALK_086944) and *fla18-2* (SALK_039619). The red bar represents the location of the nCounter NanoString probe. **(B)** *FLA18* gene expression at the primary root of the indicated genotypes. Seedlings were germinated on medium containing 1% sucrose, for 4 d, and then transferred to either sucrose-free or 4.5% sucrose-containing medium for 6 h. Gene expression was measured using the nCounter NanoString technology, in four biological replicates. Expression level is indicated in normalized counts, as calculated by the nSolver platform using *GAPC, UBQ10, EF1a, F-BOX*, and *AP2* as reference genes. **(C)** Seedlings of the indicated genotypes were grown on sucrose-free MS medium for 4 days and then, transferred to either new sucrose-free MS medium or MS medium supplemented with 4.5% sucrose, for an additional 10 days. Whole seedlings (upper panel), lateral root tips (LR, middle panel) and primary root tips (PR, lower panel) were documented. Total lateral root length **(D)** and diameter **(E)** were measured 7 days after the transfer to restrictive conditions (MS plus 4.5% sucrose). Error bars represent SE. The results were analyzed through JMPpro13 for statistical analysis, applying Tukey’s HSD test (**B**; ***p < *0.0001*) or a non-parametric comparison for all pairs using the Steel-Dwass Method (**D,E**; *p* < *0.0001*). D and E, *n* = 10. Scale bars = 1 cm (**C**; upper panel) and 1 mm (**C**; middle and lower panel).

To investigate the role of *FLA18* during root elongation, two independent T-DNA insertion lines were obtained, SALK_086944 named *fla18-1* and SALK_039619 named *fla18-2*, both carrying a T-DNA insertion in the first exon of the *FLA18* coding sequence (Figure 1A and Supplementary Figure 4A). Homozygous lines were identified by genotyping. The expression of *FLA18* in both *fla18-1* and *fla18-2* was examined by RT-PCR analysis using *FLA18*-specific primers localized downstream (3′) of the T-DNA insertion site and *TUBULIN* as a reference gene. According to the RT-PCR analysis both *fla18-1* and *fla18-2* appeared to be knock-down alleles, as both exhibited very low expression compared to the wild-type, but neither completely abolished the *FLA18* gene expression (Supplementary Figure 4B). The reduced expression level in *fla18-1* was verified by nCounter NanoString analysis, using a *FLA18*-specific probe targeting the sequence localized downstream of the T-DNA insertion site (Figure 1B). An approximate 10-fold reduction in *FLA18* gene expression was measured in the *fla18-1* mutant background as compared to the wild type (Figure 1B).

To study the possible role of FLA18 in the context of root elongation, the mutant lines were grown under either ambient conditions [Murashige and Skoog (MS) medium with no sucrose added] or restrictive conditions (MS supplemented with 4.5% sucrose). These restrictive conditions have been previously shown to act as sensitizing conditions that may serve to uncover perturbations in cell wall deposition during root elongation (Hauser et al., 1995; Schindelman et al., 2001; Xu et al., 2008; Basu et al., 2016). When grown under ambient conditions, the mutant lines resembled the wild type (Figure 1C), except from a slight increase in PR elongation rate and final length (Supplementary Figures 2, 5). When grown under restrictive conditions, the LRs of both *fla18-1* and *fla18-2* displayed an approximate 30% reduction in length compared to the wild type (Figure 1D and Supplementary Figure 6). Furthermore, a significant increase of about 30% in LR width was also detected (Figure 1E). It is important to note that the *fla18* LR phenotype can be modified by different MS or agar types used in the media. Different batches (lot numbers) of the same product, MS or gelling agent, showed different LR phenotype in *fla18* background, ranging from short and swollen to indistinguishable from the wild-type (data not shown). No specific difference in nutrient composition could be associated with this effect on the *fla18* root phenotype. Nonetheless, under the experimental conditions mentioned above, this phenotype was observed in multiple independent experiments. Transgenic lines (T3) expressing the *FLA18* coding sequence driven by the 35S promoter, complemented the LR phenotype of the *fla18-1* allele when grown on high-sucrose containing medium (Supplementary Figure 7). Note that complementation line #2, which is a strong suppressor of the *fla18* root phenotype, seem to display reduced LR density as compared to the wild-type. It is noteworthy, that despite the fact that *FLA18* gene expression was detected in other developmental contexts additional to the root, no other phenotypes were detected for either *fla18-1* or *fla18-2*. In summary, these observations identify a new role for FLA18 in the regulation of root elongation and suggest that it may differentially affect root elongation in PR vs. LR under different growth conditions.

### The *fla18* Mutant Background Enhances the Phenotype of *sos5* in Primary root, Lateral Root, and the Shoot Under Restrictive Conditions

**FIGURE 2:**
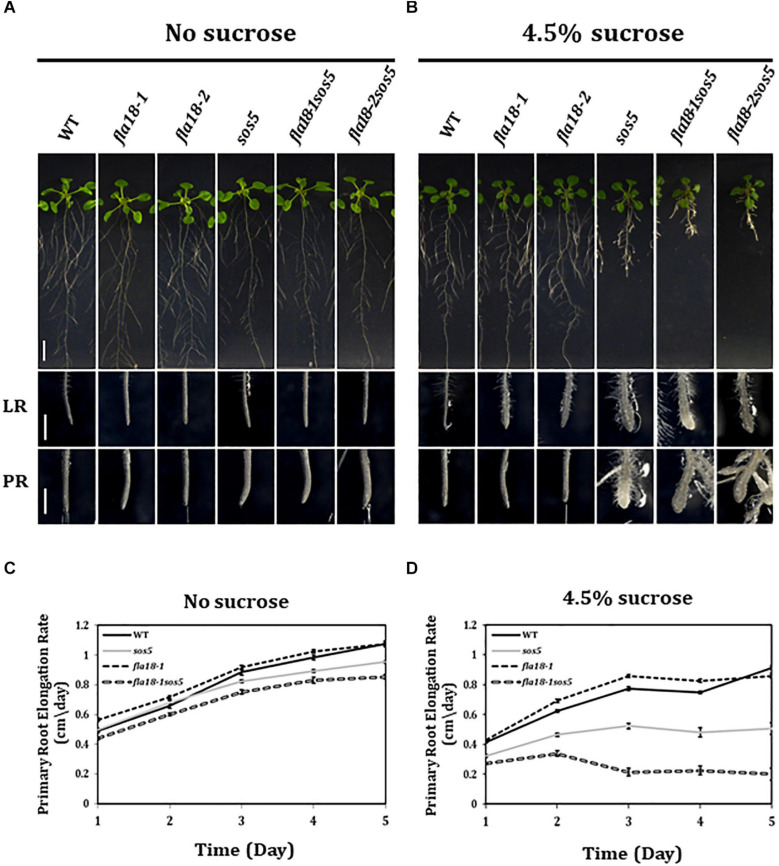
*fla18sos5* double mutants impart a synergistic effect on root elongation. **(A,B)** Seedlings of the indicated genotypes were grown on sucrose-free MS medium for 4 days and then transferred to either fresh sucrose-free medium (**A**; permissive conditions) or medium containing 4.5% sucrose (**B**; restrictive conditions), for an additional 7 days. Whole seedlings (upper panel), lateral root tips (LR, middle panel) and primary root tips (PR, lower panel) were documented. Note, the synergistic effect of the double mutant on both PR and LR elongation and swelling. **(C,D)** Seedlings of the indicated genotypes were grown on MS medium for 4 days and then transferred to sucrose-free MS medium **(C)** or MS medium plus 4.5% sucrose **(D)**. Root length was measured daily and elongation rate was calculated; *n* = 10 (*fla18-1*; *n* = 5). Error bars represent SE. Scale bars = 1 cm (**A,B**; upper panel) and 1 mm (**A,B**; middle and lower panel).

The finding that both *fla18* and *fla4/sos5*, herein referred to as *sos5*, display perturbation of LR elongation suggests a non-redundant role in this developmental context under restrictive conditions of high-sucrose containing medium. Taking into account the phylogenetic distance between these two FLA-encoding genes this might be expected (Supplementary Figure 1). We therefore generated the *fla18-1sos5* and *fla18-2sos5* double mutants to study the relationship between these two FLA-encoding genes in the context of root elongation. The results with both *fla18* alleles were the same (Figure 2 and Supplementary Figure 9, data not shown) and hence quantification is presented only for *fla18-1sos5*. The severity of the *fla18sos5* phenotype was modified in response to different growth conditions. Under ambient conditions (MS media with no sucrose), no root swelling was observed, but slight differences in the rate of root elongation were detected (Figure 2). As previously noted (Figure 1), the rate of *fla18-1* PR elongation was slightly higher as compared to the wild type (Figure 2C) and the final root length was slightly longer (Supplementary Figure 8). At the same time, *sos5* and to a greater extent *fla18-1sos5*, displayed a reduced PR elongation rate, as compared to the wild type (Figure 2C and Supplementary Figure 8). When grown on MS media supplemented with 1% sucrose, the *fla18sos5* LRs and PRs displayed swollen root tips, unlike any of the single mutants or the wild type (Supplementary Figure 9). Last, under restrictive conditions (MS supplemented with 4.5% sucrose), both wild type and *fla18-1* displayed normal root growth, while *sos5* displayed a short and swollen root phenotype in both LR and PR, consistent with previous findings (Figure 2B; Shi et al., 2003; Xu et al., 2008; Basu et al., 2016). Interestingly, under these conditions, the *fla18sos5* double mutants displayed a more severe perturbation of anisotropic root growth, as indicated by the very short and swollen root phenotype observed in both PR and LRs (Figure 2B). Quantification of PR elongation rate, following the transfer to restrictive conditions, demonstrated that the *fla18-1sos5* roots display a much more severe response to the restrictive conditions, compared to all other genotypes examined in this experiment (Figures 2C,D and Supplementary Figure 8). Altogether, the *fla18sos5* double mutant displays a severe effect on both PR and LR elongation, as compared to either of the single mutants or the wild-type, when grown on mild- or high-sucrose containing media.

**FIGURE 3:**
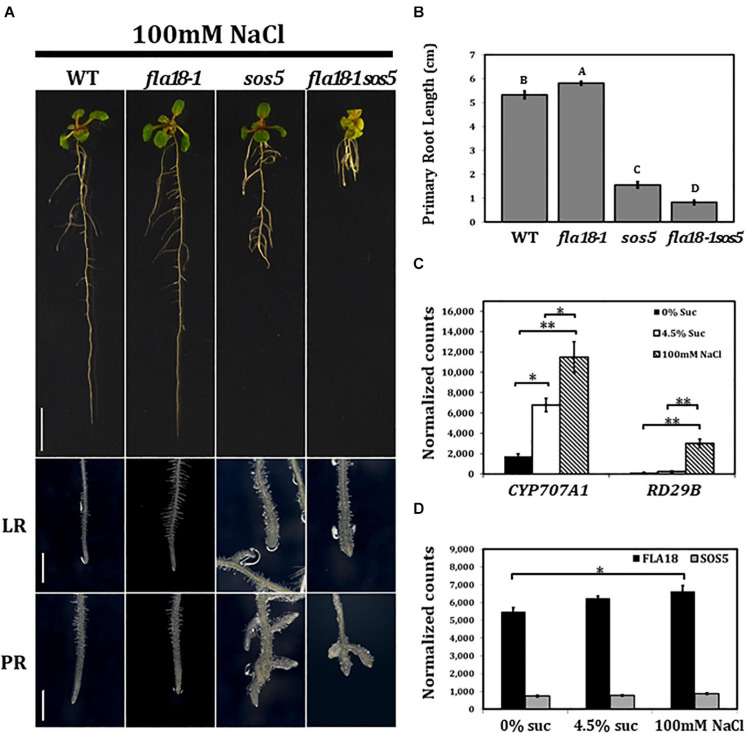
*fla18sos5* double mutants impart a synergistic effect on root elongation under salt stress conditions. Seedlings of the indicated genotypes were grown on MS medium containing 1% sucrose, for 4 days, and then transferred to medium containing 1% sucrose and 100 mM NaCl for an additional 7 days. **(A)** Whole seedlings (upper panel), lateral root tips (LR, middle panel) and primary root tips (PR, lower panel). **(B)** Measurements of primary root length were conducted 7 d after seedlings were transferred to restrictive conditions (MS supplemented with 1% sucrose and 100 mM NaCl). *n* = 6. **(C,D)** Gene expression of the indicated genes was examined in the PR of wild type seedlings germinated on medium containing 1% sucrose, for 4 days, and then transferred to medium containing either no sucrose, 4.5% sucrose or 1% sucrose + 100 mM NaCl, for 6 h. Gene expression was measured using nCounter NanoString technology, in four biological replicates. Expression level is indicated by normalized counts, as calculated by the nSolver platform using *GAPC*, *UBQ10*, *EF1a*, *F-BOX*, and *AP2* as reference genes. The results were analyzed using JMPpro13 for statistical analysis and analyzed using Tukey’s HSD (honestly significant difference) test (*p < *0.05*,**p < *0.001*). Scale bars = 1 cm (**A**; upper panel) and 1 mm (**A**; middle and lower panel).

The effect of the different genotypes on root elongation was examined also under salt stress conditions (Figure 3). As mentioned above, *sos5* is a conditional mutant that displays root growth arrest and root tip swelling when grown on high-sucrose or high-salt containing media (Shi et al., 2003; Xu et al., 2008; Xue and Seifert, 2015; Basu et al., 2016). In the current study, when grown on MS supplemented with 100 nM NaCl, roots of the *fla18-1* allele looked similar to the wild type, yet a slight but significant increase in PR length could be detected (Figures 3A,B). Unlike *sos5*, no phenotype was detected in *fla18* LRs (Figure 3A). Short and swollen LRs in the *fla18* mutants were observed only on high-sucrose and not on high-salt containing media (Figures 1–3). However, the most prominent phenotype was that of the *fla18-1sos5* double mutant. When root elongation of the *fla18-1sos5* seedlings was examined under salt-stress conditions, a significant reduction in PR length was observed of about 84% compared to either *fla18-1* or the wild-type and about 52% compared to the *sos5* single mutant (Figure 3B). Interestingly, under these conditions, *fla18-1sos5* displayed also a small and chlorotic shoot phenotype, that was not detected in any of the other genotypes examined (Figure 3A).

To investigate the effect of the different growth conditions on *FLA* gene expression in roots, total RNA was extracted from roots of 4-days old seedlings, 6 h after the transfer from ambient to either of the examined conditions, and subjected to nCounter NanoString analysis. After 6 h no phenotype could be observed, yet stress response could already be detected, as indicated by the induced expression of *RESPONSIVE TO DESICCATION 29B* (*RD29B*) and *CYTOCHROME P450, FAMILY 707, SUBFAMILY A, POLYPEPTIDE 1* (*CYP7071A*; Ingram and Bartels, 1996; Xiong et al., 2002; Nambara and Marion-Poll, 2005; Seifert et al., 2014). At this time point, the expression of *RD29B* in wild type was 20-fold higher under salt-stress conditions compared to ambient conditions (Figure 3C), but was not significantly altered in roots transffered to high-sucrose containing medium (Figure 3C). In contrast, *CYP7071A*, which is involved in ABA catabolism, showed an approximate fourfold increase in roots grown on high sucrose-containing medium and an approximate sixfold increase in roots grown on high salt-containing medium, as compared to ambient conditions (Figure 3C). Neither *FLA18* nor *SOS5* gene expression was significantly modified by the abiotic, high-sucrose or high-salt, stress conditions (Figure 3D). To learn more more about the interaction between *FLA18* and *SOS5*, we followed the expression of each of these genes in the different mutant backgrounds (Supplementary Figure 10). As expected, *FLA18* gene expression was significantly reduced in *fla18-1* and *fla18-1sos5* mutant background, compared to the wild-type. Interestingly, a mild but significant increase in *FLA18* gene expression could be detected in *sos5* mutant background when grown under either permissive (MS with no-sucrose added) or restrictive conditions (MS supplemented with 4.5% sucrose; Supplementary Figure 10). For *SOS5*, a significant twofold decrease was detected in *sos5* and *fla18-1sos5* mutant background, as compared to the wild-type. This was somewhat surprising as the *sos5-2* allele used in this study is a T-DNA insertion line that was previously described as a null allele (Xu et al., 2008; Harpaz-Saad et al., 2011; Griffiths et al., 2014; Seifert et al., 2014; Xue and Seifert, 2015; Basu et al., 2016; Xue et al., 2017). One explanation could be that the probe recognition site in this case is localized upstream of the T-DNA insertion site. Nonetheless, a minor increase in SOS5 gene expression could be detcetd in *fla18-1* mutant background, which was statistically significant under restrictive conditions (Supplementary Figure 10). Altogether, these results pinpoint to the different mechanisms induced under different stress conditions and suggest that FLA18 and FLA4 each plays a unique vs. partially overlapping roles during plant development under different growth conditions.

### The *fla18* and *fla18sos5* Root Phenotype Is Suppressed by ABA and Hypersensitive to Inhibition of ABA Synthesis

**FIGURE 4:**
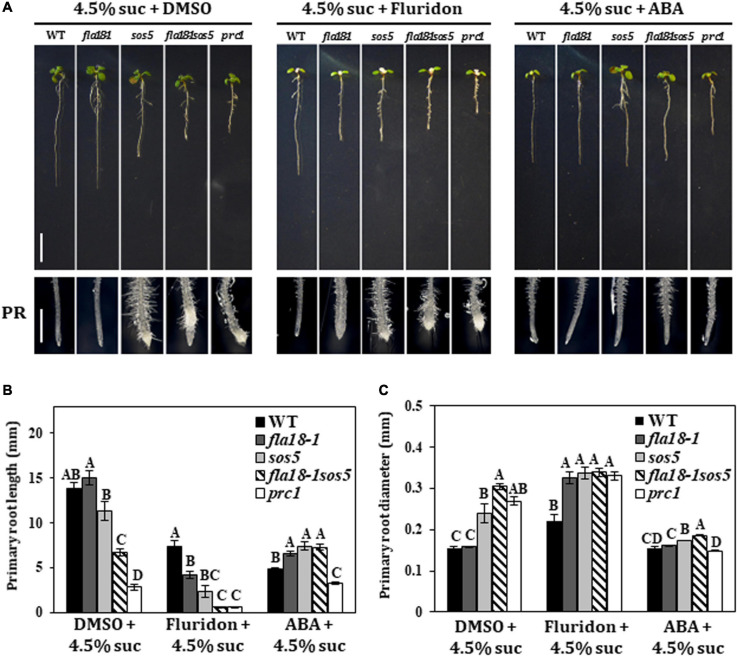
Root elongation in *fla18* and *fla18sos5* is suppressed by ABA and hypersensitive to the ABA-synthesis inhibitor, Fluridon. **(A)** Seedlings of the indicated genotypes were grown on MS medium with 1% sucrose, for 5 days, and then transferred to medium containing 4.5% sucrose and either 5 mM ABA, 5 mM Fluridon or similar volume of DMSO, the ABA/Fluridon solvent. **(B,C)** Measurements of primary root length **(B)** and diameter **(C)** were conducted 48 h after seedlings were transferred to new media. The results were analyzed using JMPpro13 for statistical analysis and run through Tukey’s HSD test (*p* < *0.05*); *n* = 6. Error bars represent SE. Scale bars = 1 cm (**A**; upper panel) and 1 mm (**A**; lower panel).

Previous studies demonstrated that FLA4/SOS5 functions as a positive regulator of cell wall deposition by modulating ABA signaling (Seifert et al., 2014). To examine the potential interaction between FLA18 function and ABA, we used exogenous ABA treatment and Fluridon, an inhibitor of the carotenoid pathway leading to ABA synthesis (Nakamura and Asami, 2014). Seedlings were grown for 5 days under ambient conditions and then transferred to medium containing either 4.5% sucrose (DMSO that served as the Fluridon solvent was added as a control), or 4.5% sucrose with the addition of ABA or Fluridon, for two additional days (Figure 4). As previously described, on MS supplemented with high-sucrose concentration, the PR of the wild type and *fla18-1* remained unswollen, while the PR of *sos5* and *fla18-1sos5* was short and swollen (Figure 4A). The same can be seen for the PR of *procuste1* (*prc1*), a mutant in *CESA6* encoding for the cellulose synthase catalytic subunit that served as a positive control for this set of experiments (Figure 4A). When exposed to ABA, the root length of the wild-type was shorter, as compared to control with no-ABA added. Interestingly, PR length of all *fla*-mutants was significantly longer than the wild type (Figure 4B). In addition, the swollen root phenotype of *sos5*, *fla18-1sos5* and *prc1* was suppressed (Figure 4C). On the other hand, Fluridon treatment, which also results in shorter wild-type PR as compared to control conditions, led to a reduction in PR length in all examined genotypes, as compared to the wild-type (Figure 4). *fla18sos5* and *prc1* displayed the most severe perturbation in root elongation in the presence of Fluridon (Figure 4B). This was accompanied by significant PR-swelling in all genotypes apart from the wild type (Figure 4C). These findings were further supported by the transgenic lines (T2) expressing the coding sequence of *FLA18*, driven by 35S promoter, in *fla18* mutant background. *FLA18* overexpression reversed the Fluridon hypersensitivity of the *fla18* mutants (Supplementary Figure 11). Complementation of the *fla18* phenotype was evident in both PR length and diameter and was documented for three independent lines (Supplementary Figure 11). To further examine the relationship between ABA and the FLA proteins during root development we followed the expression of *CYP707A1*, encoding an ABA 8’-hydroxylase employed in ABA catabolism and *C-TERMINAL DOMAIN PHOSPHATASE LOCI 1* (*CPL1*), encoding a negative regulator of ABA-induced genes. The expression of *CPL1* remained un-altered in all *fla* mutants examined, as compared to the wild type (Supplementary Figure 12). However, the level of *CYP707A1* expression was slightly but significantly induced in all *fla*-mutants examined, under permissive conditions, as compared to the wild-type. Under restrictive conditions, the levels of *CYP707A1* expression was slightly reduced in both single mutants and slightly increased in the *fla18-1sos5* double mutant background, as compared to the wild type (Supplementary Figure 12). To summarize, these observations support a role for FLA18 in both LR and PR elongation. Moreover it suggests that, similar to FLA4/SOS5, FLA18 may also display cross-talk with ABA through a yet to be identified mechanism.

### *fla18sos5* Is Hyper-Sensitive to the Cellulose Synthase Inhibitor, Isoxaben

**FIGURE 5:**
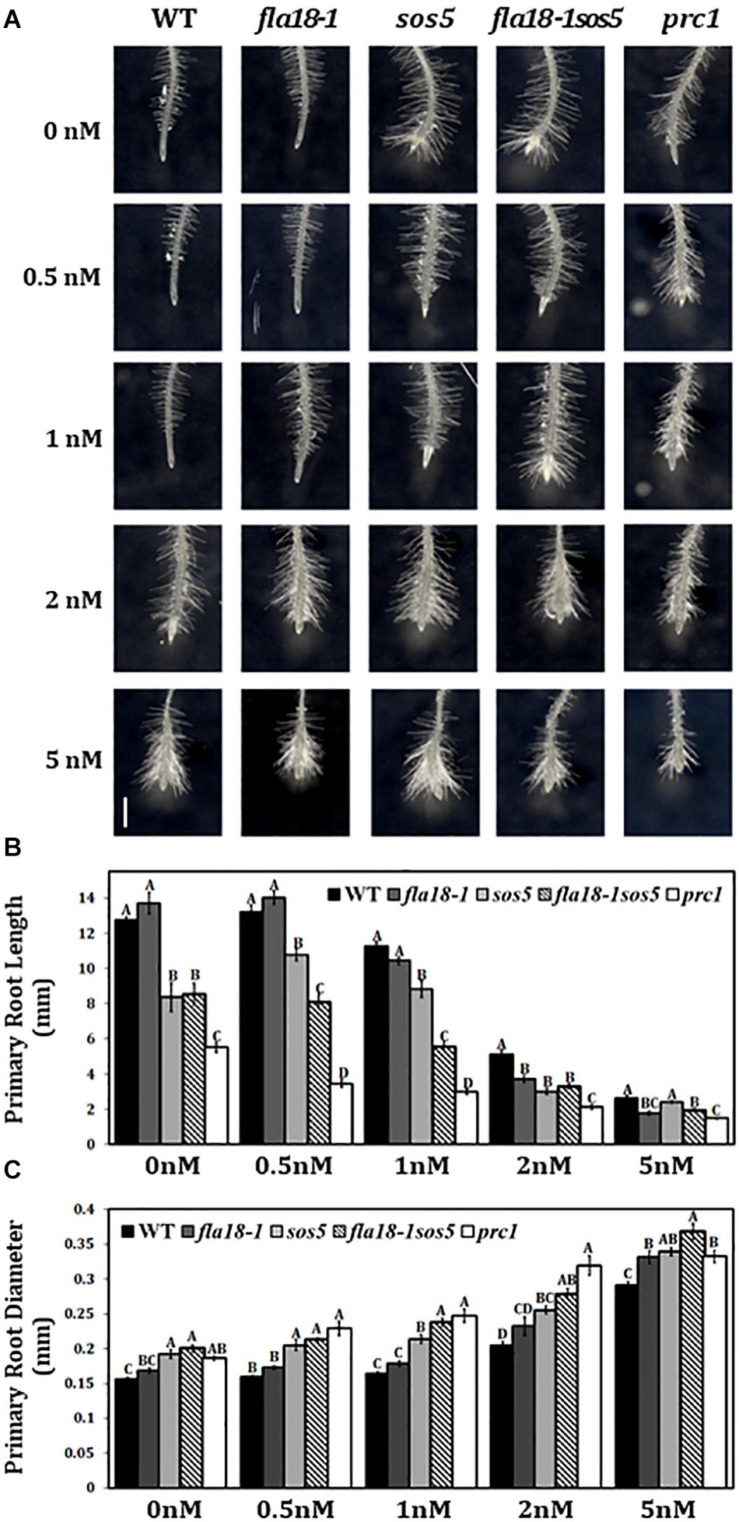
*fla18* and *fla18sos5* are hypersensitive to the cellulose synthase inhibitor, Isoxaben. **(A)** Primary root tips of seedlings of the indicated genotypes were grown on MS medium with 1% sucrose for 4 days and then transferred to medium containing 1% sucrose and the indicated concentration of isoxaben, for 48 h. **(B,C)** Measurements of PR length **(B)** and diameter **(C)** at the end of the experiment. The results were analyzed using JMPpro13 for statistical analysis and run through Tukey’s HSD test (*p* < *0.05*). Error bars represent SE. PR length: *n* = 6 (*prc1*; *n* = 5). PR diameter: *n* = 4. Scale bar = 1 mm **(A)**.

Various cell wall mutants, including *sos5* and *prc1*, have been shown to be hypersensitive to the cellulose synthase inhibitor, Isoxaben (Desprez et al., 2002; Fridman et al., 2014; Basu et al., 2016). To investigate the effect of the *fla18* mutation on cell wall properties, we examined the sensitivity of root elongation to Isoxaben in *fla18-1* and *fla18-1sos5* mutant background. Seedlings were grown for 4 days under ambient conditions and then transferred to MS medium supplemented with 1% sucrose and increasing Isoxaben concentrations for an additional 48 h. *sos5* and *prc1*, served as positive controls for this assay. The *fla18* PR is hypersensitive to Isoxaben as reflected by the reduction in PR length as well as the increase in PR tip width compared to the wild type. The *fla18sos5* double mutant, proved even more hypersensitive to isoxaben, as compared to wild-type and the single mutants (Figure 5). This was evident by the shorter root length and increased radial swelling observed at lower concentrations of Isoxaben. The effect of isoxaben on *fla18sos5* PR length was already observed at 0.5 nM, a lower concentration compared to both wild type and the single mutants (Figure 5B) while the effect on the root diameter was only observed at 1 nM isoxaben (Figure 5C).

Previous studies done on various cell wall mutants have been able to show that in some cases, perturbation of cell wall deposition can lead to ectopic lignin deposition. Phloroglucinol is a histochemical stain that specifically detects 4-O-linked hydroxy-cinnamyl aldehydes, which form part of the lignin polymer (Supplementary Figure 13; Pomar et al., 2002). The mutants *prc1* and *sos5* served as positive controls as they were previously shown to display ectopic lignin deposition stained by phloroglucinol (Desprez et al., 2002; Caño-Delgado et al., 2003; Denness et al., 2011; Basu et al., 2016). Phloroglucinol stain demonstrated ectopic lignin deposition also in *fla18sos5* (Supplementary Figure 13). Altogether, these results suggest that FLA18 may affect root elongation through modification of cell wall deposition. Further research will be required to test this hypothesis.

## Discussion

In plants, organ growth occurs as a result of highly synchronized cell expansion of numerous cells simultaneously. To enable growth without protoplast rupture, cell wall loosening and reconstruction must occur in a tightly regulated manner. In the current study, we used tissue specific co-expression analysis to uncover new gene-products involved in the regulation of root elongation. This led to the identification of FLA18, a fasciclin-like arabinogalactan protein, as a new regulator of PR and LR elongation in *Arabidopsis*. The expression of *FLA18* suggests various roles in different developmental contexts along the course of plant development. Yet, the most prominent phenotype detected was the modification of root architecture by differential regulation of LR vs. PR growth in response to different growth conditions.

The FLA proteins are a sub-family of extra-cellular arabinogalactan-proteins (AGPs) characterized by the presence of one or two fasciclin (FAS) domains (Schultz et al., 2000, 2002; Johnson et al., 2003; Seifert and Roberts, 2007; Showalter et al., 2010). The FAS domains are 110 to 150 amino acids long, they share low overall sequence similarity, but possess two highly conserved regions of approximately 15 amino acids each, and a conserved central YH motif (Johnson et al., 2003; Seifert, 2018). FLA proteins were identified in all kingdoms including animals, yeast, bacteria, algae, lichens, and seed plants (Elkins et al., 1990; Huber and Sumper, 1994; Kawamoto et al., 1998; Gaspar et al., 2001; Paulsrud and Lindblad, 2002; Johnson et al., 2003; Novakoviæ et al., 2018; Yeats et al., 2018; Shafee et al., 2020). They were shown to be highly glycosylated and frequently predicted to have a glycosylphosphatidylinositol (GPI)-anchor that attaches them to the outer leaflet of the plasma membrane, facing the extra-cellular matrix (Schultz et al., 2002; Johnson et al., 2003; Seifert and Roberts, 2007; Ellis et al., 2010; Basu et al., 2016; Xue et al., 2017; Yeats et al., 2018). Proteins containing FAS domains, from a broad spectrum of organisms, have been shown to function as adhesion molecules (Elkins et al., 1990; Huber and Sumper, 1994; Kawamoto et al., 1998; Schultz et al., 2000).

The *FLA* gene-family in *Arabidopsis* is composed of 21 genes (Supplementary Figure 1; Schultz et al., 2000; Johnson et al., 2003; MacMillan et al., 2010; Costa et al., 2019; Shafee et al., 2020). He et al. (2019) revised this number and suggested there are only 20 *FLA* genes in *Arabidopsis*. The biological function of most *FLA*-encoded proteins remained largely unknown. Partly, because of high levels of functional redundancy and the fact that mutant analysis uncovered very few phenotypes so far. Of the limited number of *FLA* genes with established function, FLA4, also known as SOS5, was also shown to affect cell wall deposition. The *sos5* mutant displays short and swollen roots under restrictive conditions of high-sucrose or high-salt containing media. This phenotype was associated with perturbation of the middle lamella required for cell-cell adhesion (Shi et al., 2003; Xu et al., 2008; Xue and Seifert, 2015; Basu et al., 2016; Xue et al., 2017). The *sos5* mutant displays also a mild-swelling of the hypocotyls of dark-grown seedlings and perturbation of seed mucilage organization (Xu et al., 2008; Harpaz-Saad et al., 2011; Griffiths et al., 2014, 2016; Basu et al., 2016). *FLA11* and *FLA12*, which are expressed during xylem cell differentiation, were assigned a function in secondary cell wall deposition. The *fla11fla12* double mutant displays reduced cellulose content, alteration in cellulose microfibril angle, reduced tensile strength and reduced tensile modulus of elasticity. Similar results were obtained also for *FLA11* and *FLA12* orthologs in other species like cotton, poplar, eucalypt and hemp (Huang et al., 2008; MacMillan et al., 2015; Bygdell et al., 2017; Guerriero et al., 2017). Recent study, identified *FLA16* as an additional player required for secondary cell wall deposition during stem elongation in *Arabidopsis*. The presented data, demonstrate that he *fla16* mutant displays reduced stem length, alteration in stem biomechanical properties and reduced cellulose levels suggesting a role in secondary cell wall deposition (Liu et al., 2020). FLA3 was assigned a function in microspore development. *FLA3* RNA interference lines displayed reduced intine cell wall synthesis and reduced calcofluor stain for glycans in aborted pollen grains (Li et al., 2010). Hence multiple FLA proteins were assigned function in cell wall deposition or organization. In addition to the FLA proteins mentioned above, mutant analysis and gene expression studies have suggested that FLA1 plays a role in early events of shoot regeneration in tissue culture (Johnson et al., 2011). The phenotypic analysis of mutants in *FLA9* suggest it is essential to prevent seed abortion (Cagnola et al., 2018). The function of the other *FLA*-encoding genes, fourteen in *Arabidopsis*, is yet to be identified.

The *Arabidopsis FLA* genes were classified into four groups based on the number of FAS domains (one or two), the number of AGP domains (one or two), and whether they contain a predicted GPI-anchor modification site (Johnson et al., 2003; MacMillan et al., 2010). Phylogenetic studies suggest that these four groups are maintained throughout the evolution of land plants (Costa et al., 2019; He et al., 2019; Shafee et al., 2020). *FLA18* was classified as part of group B which contains *FLA15*, *FLA16*, *FLA17* and *FLA18*. Group B FLAs have two FAS1 domains separated by an AGP-region and are not predicted to contain a GPI-anchor. Recent study by Liu et al. (2020), assigned function to the first group B-*FLA*, *FLA16*. The *fla16* mutant displays short stems, reduced stem diameter, reduced stem pith cell number, reduced stem cellulose levels, and modification of stem biomechanical properties. These suggest that FLA16 plays a role as part of the mechanism regulating secondary cell wall deposition and cell wall integrity during stem growth. This was further supported by the localization of FLA16-fusion protein, driven by the endogenous promoter, to the cell wall and plasma membrane-wall interface of cells producing secondary cell wall like fiber and xylem cells. Interestingly, etiolated seedlings of the *fla16* mutant display reduced hypocotyl length as compared to the wild type and hypersensitivity to the cellulose synthase inhibitor, Isoxaben. Given that secondary cell wall cellulose synthesis is not affected by isoxaben (Watanabe et al., 2018) this suggests a possible role also as part of the mechanism required for primary cell wall deposition in the context of dark-grown seedlings (Liu et al., 2016). In the current study, we identified *FLA18* through tissue-specific co-expression analysis using established players involved in primary cell wall deposition required for PR elongation. Given that genes involved in the same metabolic process tend to express in a transcriptionally coordinated manner (Persson et al., 2005; Ruprecht and Persson, 2012; Ben-Tov et al., 2015; Voiniciuc et al., 2015a, b, 2018) this suggests that FLA18 may play a role in this process. Further support for this hypothesis arise from the short and swollen LR phenotype of the *fla18* mutants, which is a characteristic feature of various cell wall mutants, like: *CELLULOSE SYNTHASE A 6* (Fagard et al., 2000), *CELLULOSE SYNTHASE A 3* (Caño-Delgado et al., 2003), *COBRA* (Schindelman et al., 2001), and others. Moreover, the double mutants *fla18-1sos5* and *fla18-2sos5* display a more severe perturbation of both LR and PR anisotropy, compared to each of the single mutants. Additionally, both *fla18* and *fla18sos5* are hypersensitive to the cellulose synthase inhibitor, Isoxaben. Altogether, these results support a role for FLA18, a group B-FLA, as part of the complex mechanism required for primary cell wall deposition during LR and PR elongation. Further studies will be required to prove whether FLA18 is indeed part of the mechanism employed in cell wall deposition.

It is interesting to note, that in most cell wall mutants, the perturbation of root anisotropy can be observed in both PR and LRs. This includes mutants in genes required for cell wall synthesis and deposition, including: *CELLULOSE SYNTHASE A 6* (Fagard et al., 2000), *CELLULOSE SYNTHASE A 3* (Caño-Delgado et al., 2003), *CELLULOSE SYNTHASE A 1* (Williamson et al., 2001) *COBRA* (Schindelman et al., 2001), *CHITINASE LIKE 1* (Hermans et al., 2010), *CELLULOSE SYNTHASE INTERACTIVE PROTEIN 1* (Hauser et al., 1995), and others. Additionally, mutants like *fei1fei2* and *sos5*, affecting cell wall organization through a yet to be identified mechanism, also demonstrate perturbation of root growth anisotropy in both PR and LRs, under restrictive conditions (Shi et al., 2003; Xu et al., 2008; Basu et al., 2016; Xue et al., 2017). Altogether, this accumulating data, led to the hypothesis that the same set of core components is required for root elongation in both PR and LRs. To our surprise, when we examined the *fla18* mutants, perturbation of root anisotropy was observed only in LRs, when examined under restrictive conditions of high-sucrose containing medium. While the PR displays slight but consistent increase in elongation rate, under permissive conditions, as compared to the wild type. This suggests a divergence in the mechanism involved in the regulation of PR and LR elongation. Various studies have shown that PRs and LRs may display distinct growth dynamics in response to different developmental or environmental cues. For example, mild nitrate deficiency significantly enhances LR growth, but does not affect the growth of the PR (Zhang, 1998; Zhang et al., 1999; López-Bucio et al., 2003; Gruber et al., 2013). In another example, low phosphate levels result in modification of root architecture by reducing the rate of both PR and LR elongation but each responds to different phosphate concentrations (Williamson et al., 2001; López-Bucio et al., 2002; Pérez-Torres et al., 2008; Gruber et al., 2013). Similarly, accumulating data demonstrate that the plant hormone ABA has a much stronger inhibitory effect on LR growth as compared to the effect on PR growth (Signora et al., 2002; De Smet et al., 2003; Duan et al., 2013). Altogether, these results suggest that root morphogenesis can be altered by differential regulation of PR vs. LR elongation in response to different developmental and environmental signals. Moreover it suggests that FLA18 is not a core component of cell wall deposition but rather can affect PR and LR elongation in opposite ways yielding modification of root architecture.

Phenotypic analysis of *fla18*, *sos5*, and the *fla18sos5* double mutant suggests that a sub-set of FLA proteins is required to maintain root growth under different growth conditions. The genetic interaction between *fla18* and *sos5* demonstrate that in the *fla18* background the phenotype of *sos5* becomes much more severe, as compared to each of the single mutants and the wild type. Despite the phylogenetic distance between these two FLA proteins (Supplementary Figure 1), the genetic interaction between these two mutants suggests some levels of functional redundancy. This is supported by the following observations: (i) the *fla18sos5* mutant displays reduced root growth under permissive conditions in which the *sos5* root resembles the wild-type and *fla18* display slightly longer PR compared to the wild type; (ii) The double mutant displays a synergistic effect on both PR and LR elongation leading to shorter and more swollen roots, as compared to each of the single mutants and the wild type, under restrictive conditions of either high-sucrose or high-salt containing media; and (iii) a small and chlorotic shoot phenotype that can be detected when seedlings are grown on high-salt containing medium, a phenotype that cannot be detected in any of the single mutants. These results indicate that FLA18, FLA4/SOS5, and potentially additional FLA proteins (like FLA17, the closest homolog of FLA18) each plays a unique role and yet they are partially functionally redundant in various developmental contexts. Previous studies already present evidence for functional redundancy within the *FLA*-gene family. For example, MacMillan et al. (2010), found that while single-mutants in *fla11* or *fla12* exhibited a very mild phenotype, the *fla11fla12* double mutant displayed a more pronounced phenotype compared to the wild-type and either of the single mutants. Recent study, demonstrated that FLA16 is an additional player affecting secondary cell wall deposition during stem growth (Liu et al., 2020). Moreover, gene expression studies suggested that other group B FLAs like *FLA15* and *FLA18* are also highly expressed in elongating stems, suggesting additional FLAs may play a role in this context (Liu et al., 2020). It will be fascinating to see whether FLA18 functions in both primary and secondary cell wall deposition. Future research will be required in order to identify the sub-set of FLAs that function in each developmental context and the way specific vs. overlapping roles are determined.

## Experimental Procedures

### Plant Material

The Columbia (Col-0) ecotype of *Arabidopsis thaliana* was used in this study. The *fla18* alleles (*fla18-1*, SALK_086944; *fla18-2*, SALK_039619), *sos5* [SALK_125874; (Xu et al., 2008; Harpaz-Saad et al., 2011; Seifert et al., 2014; Xue et al., 2017; Basu et al., 2016; Griffiths et al., 2016)], and *prc1* (Fagard et al., 2000) mutants were obtained from the Arabidopsis Biological Resource Center (Alonso, 2003). Homozygous plants of each line were identified via PCR-based genotyping, using gene and insert-specific primers (detailed in Supplementary Table 2).

### Growth Conditions and Measurements

For growth in soil, plants were grown at 22°C in 75 μE, under long day conditions, with a light regime of 16/8 h. For growth *in vitro*, seeds were surface-sterilized, sown on bacteriological square petri dishes (120 mm x 120 mm x 17 mm, Greiner Bio-One, 688102) containing1x Murashige and Skoog (MS) medium (Tivan Biotech, MSP01-50LT) with pH adapted to 5.8 using KOH and 0.6% Phytagel (Sigma-Aldrich, P8169). Following cold treatment at 4°C, for 4 d in the dark, plants were grown for 4–7 days in an upright position, at 22°C in 75 μE, under long-day conditions, with a light regime of 16/8 h. For induction of restrictive conditions *in vitro*, plants were transferred after 4 d in the light to plates maintained in an upright position, with MS medium containing 0.6% phytagel and one of the following additions: 4.5% sucrose, 100 mM NaCl plus 1% sucrose or the indicated concentrations of Isoxaben (Sigma-Aldrich, 36138) or Fluridon (Sigma-Aldrich, 45511). Plants transferred to MS medium containing 0.6% phytagel and the corresponding solvent served as control (ambient conditions). The root images were documented using a stereo-microscope (SMZ1270, Nikon) equipped with a camera (NIKON, DS- Ri2).

### Growth Measurements

Root length was measured using Image J (FIJI) software (Schindelin et al., 2012) and the results were analyzed using the JMP pro13 or JMP pro 15 software for statistical analysis (Statistical Discovery^TM^, SAS). For the measurement of root elongation rate, the root tip of each seedling was marked daily for 5 consecutive days after their transfer to the indicated medium. Measurements of PR and LR length were conducted 7 d after the transfer to the indicated conditions. Due to large variability in LR length, in each plant, 2 of the longest roots, located in the upper third part of the root system, were selected and used for LR length measurements (Supplementary Figure 6). PR and LR diameter was measured at the widest point of the root tip, approximately 1–4 mm from the root tip.

### RT-PCR Analysis

Total RNA was isolated from the indicated tissues using a plant/fungi total RNA extraction kit (Norgen Biotek, 25800), followed by Turbo-DNase treatment (Invitrogen, AM1907). The first strand of complementary DNA (cDNA) was synthesized from 1 μg of the total RNA using SuperScript II reverse transcriptase (Invitrogen, 18064022), according to the manufacturer’s instructions. PCR products from the cDNA of the indicated genotypes (Wild type, *fla18-1*, *fla18-2*, and *fla4/sos5*) were amplified, 28 cycles for each of the reactions, using *FLA18* or *TUBULIN*-specific primers. *TUBULIN* served as a reference gene (Supplementary Table 2).

### *FLA18* Cloning and Transgenic Plants

Wild type genomic fragments comprising the promoter region of *FLA18* (2260 bp) or coding sequence region (1390 bp) were amplified by PCR (Supplementary Table 2) using the Phusion Taq polymerase (Thermo Fisher Scientific, F-549S), as described by the manufacturer. The fragments were cloned into pENTR-TOPO-D (Invitrogen, K240020). The resultant entry plasmid was used for the LR-clonase reaction (as described by the manufacturer; Invitrogen, 11791020), introducing: i) the *FLA18-*promoter sequence into the binary vector pGWB3 for the expression of a *FLA18*-promoter driven β-GUS protein; or, ii) the *FLA18-*coding sequence into the pGWB5 binary vector (Nakagawa et al., 2007). The *FLA18*-promoter-pGWB3 vector was transformed to wild-type Columbia background while the *FLA18*-CDS-pGWB5 was transformed to *fla18-1* for complementation. Transgenic plants were selected on hygromycin and independent lines were identified.

### Histochemical β-Glucuronidase (GUS) Activity Assay

Plants from three independent transgenic lines harboring the *FLA18* promoter driving GUS expression, were used for histochemical activity assay performed using 5-bromo-4-chloro-3-indolyl β-D-glucuronide sodium salt, as previously described (Twell et al., 1990). Briefly, samples were immersed in staining solution composed of 100 mM sodium phosphate buffer (pH 7.0) with 10 mM EDTA (pH 8.0), 0.5 mM potassium ferricyanide, 0.5 mM potassium ferrocyanide, 1 mM 5-bromo-4-chloro-3-indolyl-b-glucuronic acid, and 0.1% Triton X-100. The tissue was stained either for 1 h or overnight at 37°C, as indicated. Chlorophyll was removed using 95% ethanol. T2 plants from 8 independent transgenic lines were analyzed at different developmental stages, and 3 representative lines were documented using stereo-microscope (SMZ1270, Nikon).

### Phloroglucinol Staining

Phloroglucinol staining (Sigma-Aldrich, P3502) was performed according to a previously described procedure (Caño-Delgado et al., 2003). Seedlings were grown for 4 d on plates with sucrose-free MS and then transferred to restrictive conditions for 7 d growing period in the light. The seedlings were then stained for approximately 5 min with a 2% phloroglucinol-HCl solution.

### nCounter NanoString Analysis of Gene Expression

nCounter NanoString is a hybridization-based platform used to follow gene expression (Geiss et al., 2008; Amit et al., 2009). To monitor gene expression during root elongation, seedlings were grown for 4 d on sucrose-free MS and then transferred to fresh sucrose-free MS, MS supplemented with 4.5% sucrose or supplemented with 100 mM NaCl plus 1% sucrose - for 6 h. A total of 20–25 root tips were harvested and stored in −80°C. Total RNA was extracted using the Single Cell RNA Purification Kit (Norgen Biotek, 51800). The NanoString probes were designed and synthesized by NanoString Technologies (detailed in Supplementary Table 3)^1^. As reference genes we used *GAPC* (AT3G04120), *UBQ10* (AT5G53300), *EF1a* (AT5G60390) and *F-BOX FAMILY PROTEIN* (AT5G15710), *AP2* (AT5G46630). Counts were normalized using the nSolver, according to the nCounter Gene Expression Assay Manual^2^.

### Accession Numbers

GenBank accession numbers: *FLA18* (AT3G11700), *FLA4* (AT3G46550), *CYP707A1* (AT4G19230), *RD29B* (AT5G52300), *GAPC* (AT3G04120), *UBQ10* (AT5G53300), *EF1a* (AT5G60390), *F-BOX FAMILY PROTEIN* (AT5G15710), and *AP2* (AT5G46630).

## Data Availability Statement

The datasets presented in this study can be found in online repositories. The names of the repository/repositories and accession number(s) can be found in the article/Supplementary Material.

## Author Contributions

HA performed the experiments, analyzed and interpreted the data, and wrote the article with under the supervision of SH-S and assistance of the other authors. HR assisted with experimental procedures. AI-M assisted with RNA extraction and nCounter NanoString analysis. DZ did the bioinformatic analysis under the supervision of OT. All authors contributed to the article and approved the submitted version.

## Conflict of Interest

The authors declare that the research was conducted in the absence of any commercial or financial relationships that could be construed as a potential conflict of interest.

## Footnotes

1 http://www.nanostring.com

2 http://www.nanostring.com/uploads/Manual_Gene_Expression_Assay.pdf/

## Supplementary Material

Supplementary Figure 1 Evolutionary relationships between FASCICLIN-LIKE proteins in *Arabidopsis.*

Supplementary Figure 2 *FLA18* is co-expressed with genes involved in primary cell wall deposition during root elongation.

Supplementary Figure 3 *FLA18* expression pattern in *Arabidopsis* plants.

Supplementary Figure 4 *FLA18* gene expression in T-DNA insertion mutants, as compared to wild type.

Supplementary Figure 5 Primary root elongation in *fla18* mutants.

Supplementary Figure 6 Lateral root length measurements for quantification.

Supplementary Figure 7 Expression of *FLA18* suppresses the short and swollen lateral root phenotype of *fla18-1.*

Supplementary Figure 8 Primary root length of *fla18-1sos5.*

Supplementary Figure 9 The *fla18sos5* double mutant phenotype on media containing 1% sucrose.

Supplementary Figure 10 *FLA18* and *SOS5* gene expression in *fla18-1, sos5* and the double mutant *fla18-1sos5.*

Supplementary Figure 11 Expression of *FLA18* suppresses the *fla18-1* primary root hyper-sensitivity to the ABA-synthesis inhibitor, Fluridon.

Supplementary Figure 12 Expression of genes involved in ABA signaling and metabolism in *fla*-mutant background.

Supplementary Figure 13 Histochemical Staining of the *fla18-1sos5* double mutant with phloroglucinol.

Supplementary Table 1 The genes used as ‘baits’ for the tissue-specific co-expression analysis.

Supplementary Table 2 Primers used in this study.

Supplementary Table 3 Probes used in the nCounter NanoString analysis for gene expression.
