# Supplementary material for: FASCICLIN-LIKE 18 Is a New Player Regulating Root Elongation in Arabidopsis thaliana
Source: Front Plant Sci. 2021 Apr 7;12:645286. doi: 10.3389/fpls.2021.645286 (PMC8058476; doi:10.3389/fpls.2021.645286)
Supplement: Supplementary Figure 1 — Evolutionary relationships between FASCICLIN-LIKE proteins in Arabidopsis. [file Data_Sheet_1.PDF]

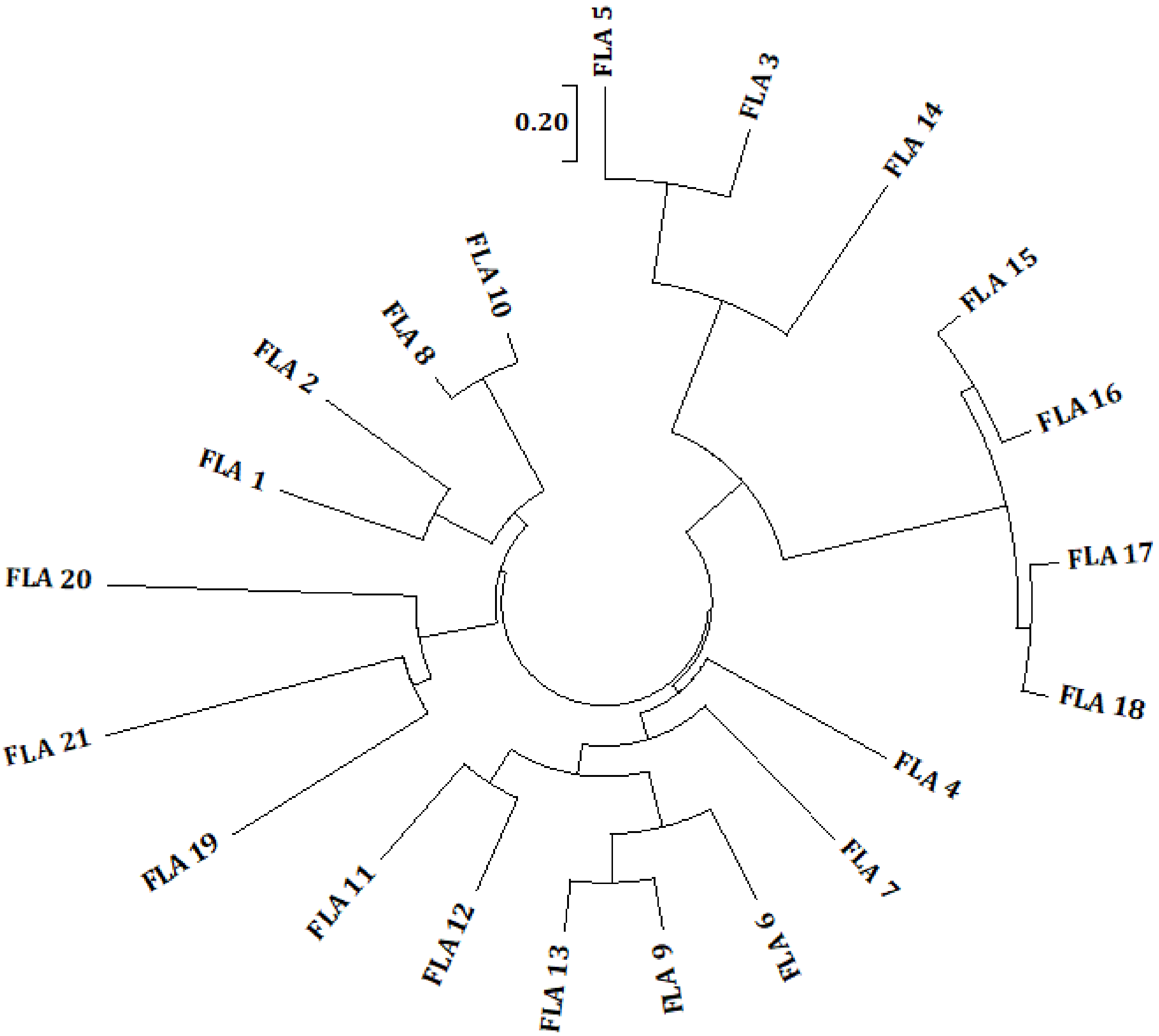

Figure S1. Evolutionary relationships between FASCICLIN-LIKE proteins in *Arabidopsis*. Evolutionary analyses were conducted using the MEGA7 software (Kumar *et al*., 2016). The evolutionary history of the FLA-gene family was inferred using the Neighbor-Joining method (Zuckerkandle *et al*., 1965; Saito and Nei, 1987). The evolutionary distances were computed using the Poisson correction method and are presented in units of the number of amino acid substitutions per-site. The analysis involved 21 amino acid sequences. All positions containing gaps and missing data were eliminated. There were a total of 157 positions in the final dataset.
The optimal tree with the sum of branch length = 9.70241807 is shown.

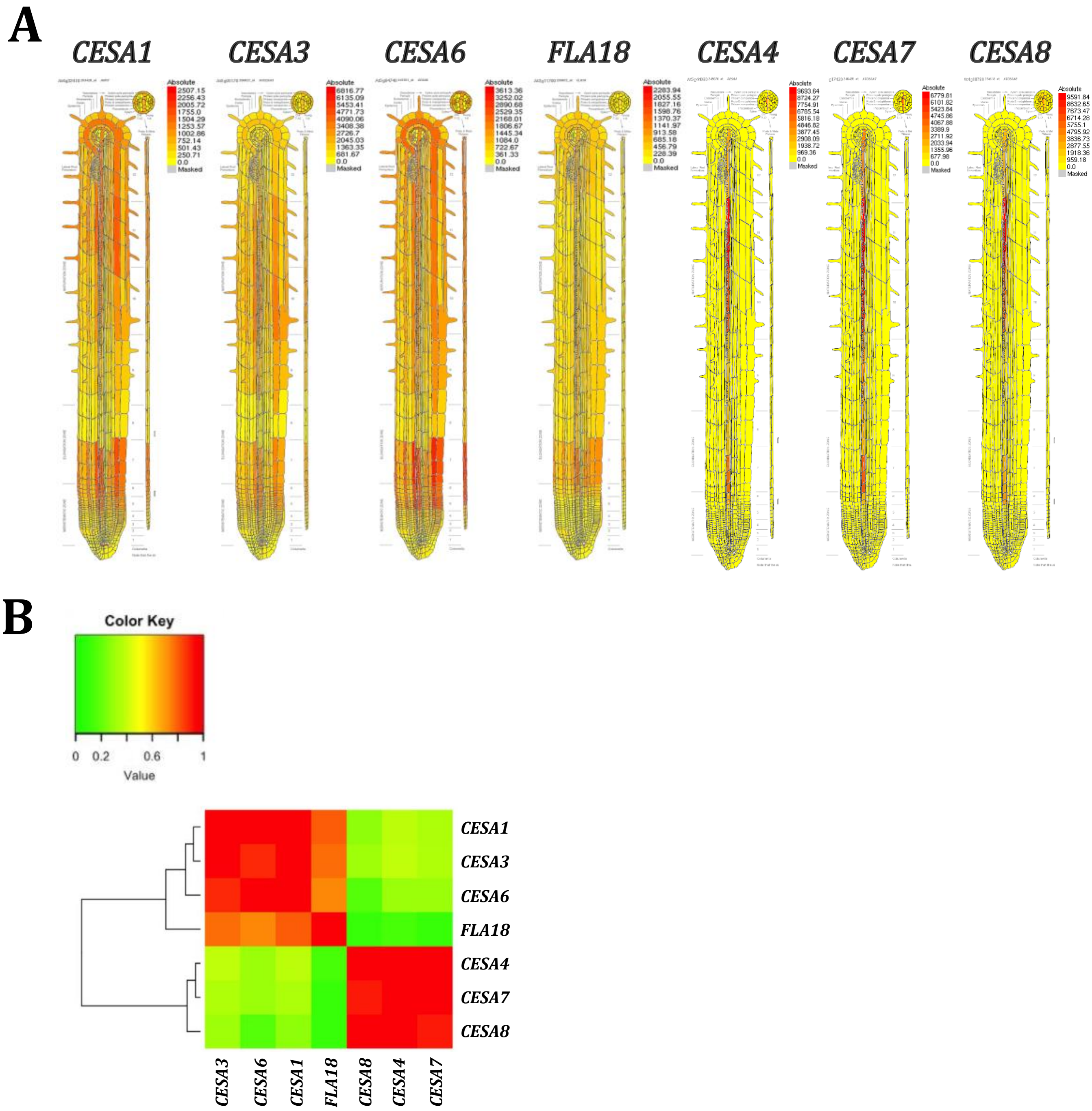

Figure S2. *FLA18* is co-expressed with genes involved in primary cell wall deposition during root elongation. (A) The expression pattern of *FLA18* and representative *CELLULOSE SYNTHASE A* (*CESA*) genes as depicted by the Bio-Array Resource eFP browser (Winter *et al.*, 2007) based on the data-set generated by (Brady *et al.*, 2007). While *CESA1*, *CESA3* and *CESA6* are required for cell wall synthesis during root elongation (and were used as 'baits' for the co-expression analysis done with CoExpNetViz http://bioinformatics.psb.ugent.be/webtools/coexpr/ ; see Table S1 for the complete 'bait' list), *CESA4*, *CESA7* and *CESA8* are required for cellulose synthesis during secondary cell wall deposition as part of cell differentiation at the vascular system. (B) Co-expression relationships between *FLA18* and representative *CESA* genes as visualized by a heat-map based on pearson correlation.

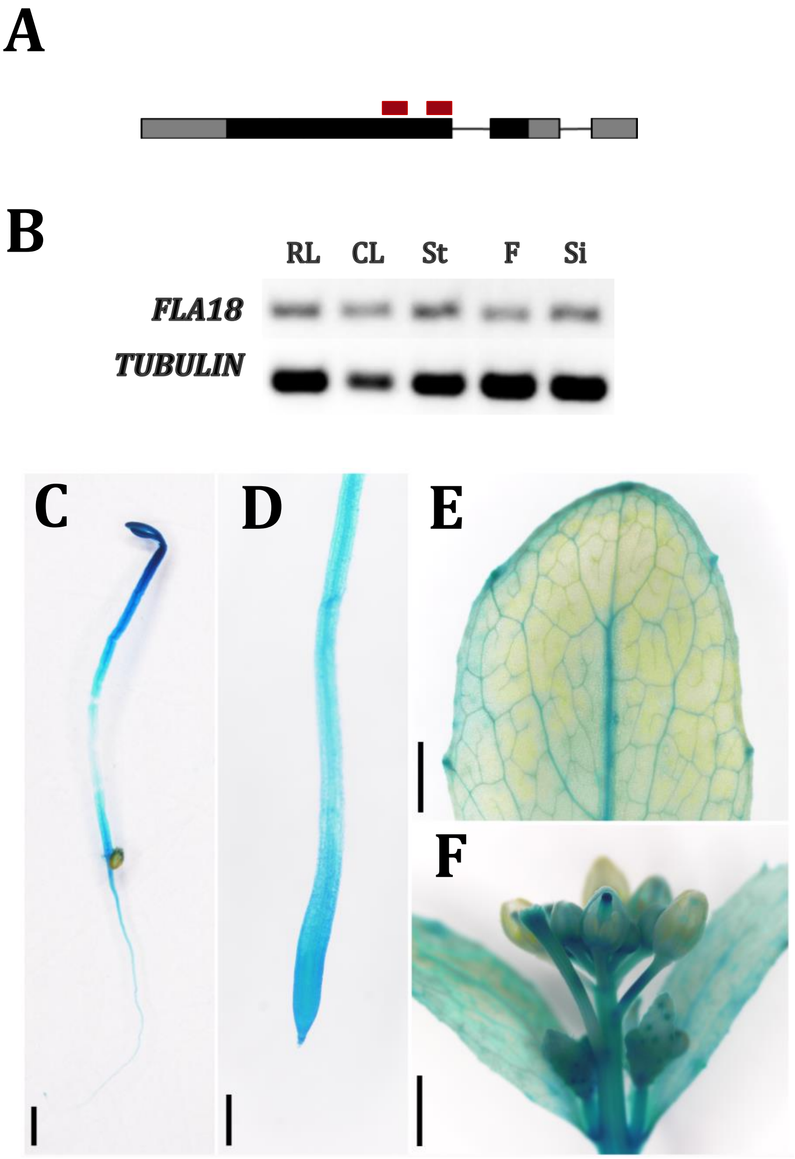

Figure S3. ***FLA18*** **expression pattern in** ***Arabidopsis*** **plants.** (A) Schematic representation of the *FLA18* gene as annotated by The Arabidopsis Information Resource. The red bars represent the locations of the diagnostic primers. (B) RT-PCR analysis was performed on total RNA from rosette leaves (RL), cauline leaves (CL), stems (ST), flowers (F) and siliques (Si) using *FLA18* or *TUBULIN* specific primers, with *TUBULIN* serving as a reference gene. (C-F) Expression of *FLA18*-promoter driven GUS in etiolated seedlings (C), primary root (D) rosette leaves (E) and inflorescence (F). Scale bars: 1 mm (C), 250 µm (D), 1 mm (E, F).

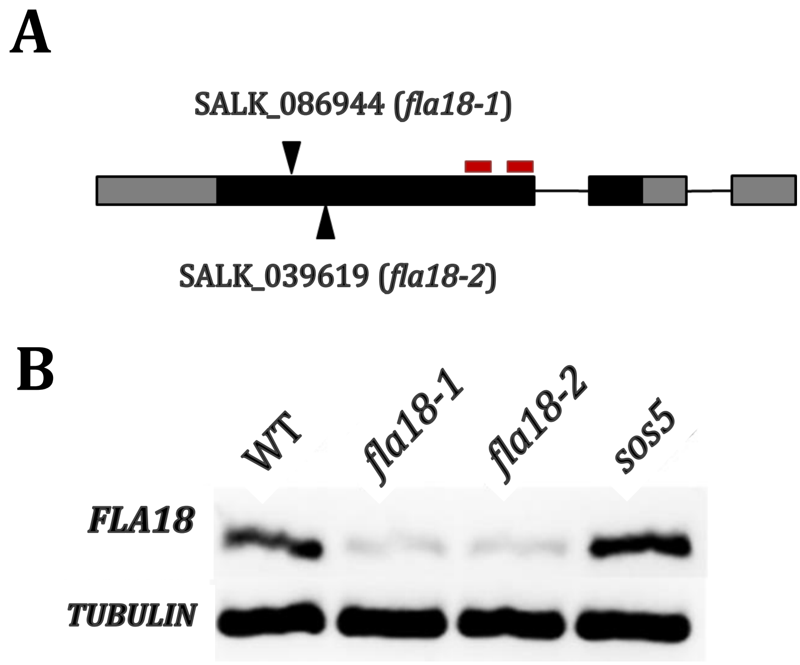

Figure S4. *FLA18* gene expression in T-DNA insertion mutants, as compared to wild type. (A) Schematic representation of the *FLA18* gene as annotated by The Arabidopsis Information Resource, pinpointing the location of the T-DNA insertion in both alleles examined, named *fla18-1* (salk_086944) and *fla18-2* (salk_039619). The red bars represent the locations of the diagnostic primers used to follow the *FLA18* gene expression. (B) *FLA18* gene expression in whole seedlings as demonstrated by RT-PCR analysis performed using *FLA18* or *TUBULIN* specific primers, with *TUBULIN* serving as a reference gene.

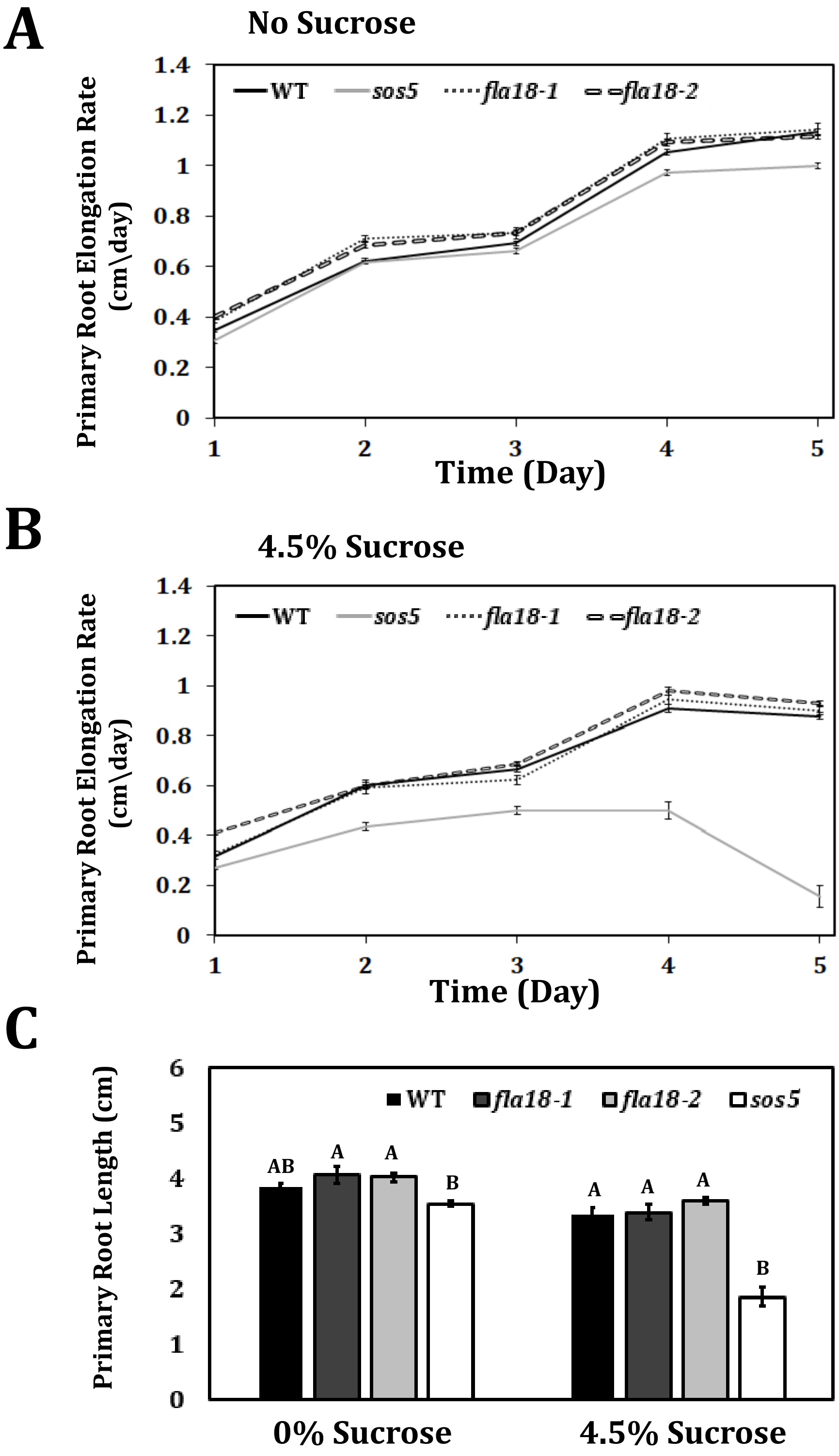

Figure S5. Primary root elongation in *fla18* mutants. Seedlings of the indicated genotypes were grown on MS medium for 4 d and then transferred to MS medium containing either (A) no sucrose, or (B) 4.5% sucrose. Primary root length was measured daily and elongation rate was calculated. (C) Primary root length was measured 5 d after the transfer to either permissive (no sucrose) or restrictive (4.5% sucrose) conditions. The results were analyzed using JMPpro13 for statistical analysis and ran through Tukey's HSD (Honestly significant difference) test (No sucrose; $p<0.05$; 4.5% sucrose; $p<0.0001$). n=10. Error bars represent SE.

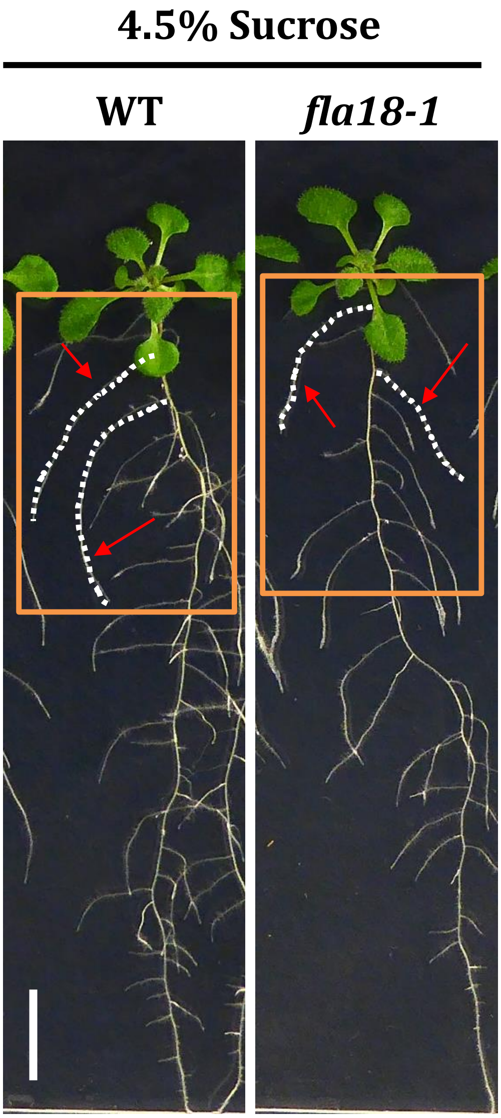

Figure S6. Lateral root length measurements for quantification. For lateral root (LR) length measurements, 2 of the longest roots, located in the upper third of the root system (orange square), were selected in each plant and used for quantification, as can be seen in the example here. Using the Image J (FIJI) software (Schindelin et al., 2012), each root was traced 5 times in order to obtain an accurate measurement of length. Root length measurements were analyzed using JMPpro15 for statistical analysis. Scale bar: 1 cm.

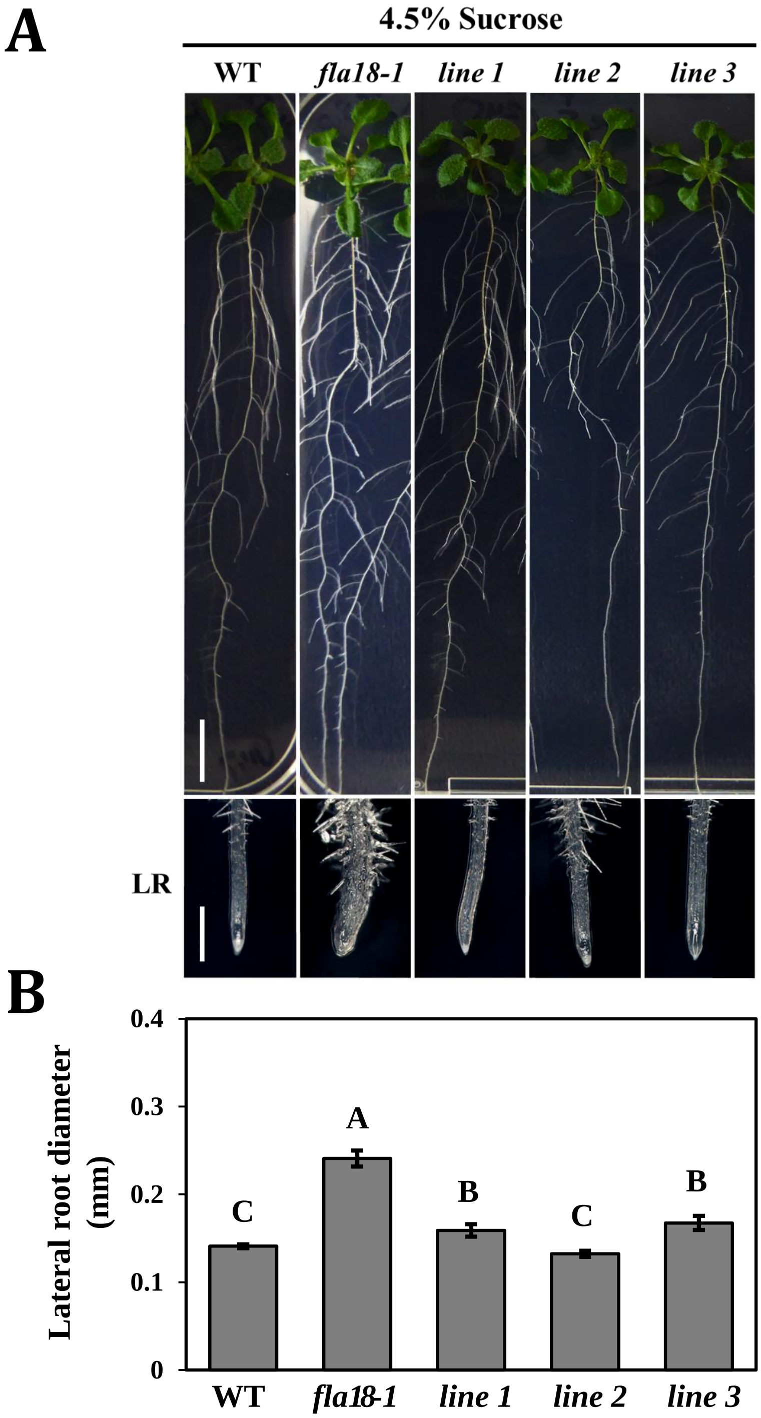

Figure S7. Expression of *FLA18* suppresses the short and swollen lateral root phenotype of *fla18-1*. (A) Seedlings of the indicated genotypes and three independent *fla18-1* mutants lines expressing the *FLA18* CDS driven by the 35S promoter were grown on MS medium with 1% sucrose for 6 d and then transferred to medium containing 4.5% sucrose for an additional 9 d. The transgenic plants are T2 generation. (B) LR diameter was measured at the end of the experiment. The results were analyzed using JMPpro15 for statistical analysis and run through Tukey's HSD (Honestly significant difference) test. {$p<0.0001$ (WT-line1; $p=0.0097$)} n=10 (*line 2*; n=8). Error bars represent SE. Scale bars: 1 cm (A; upper panel), 0.5 mm (A; lower panel).

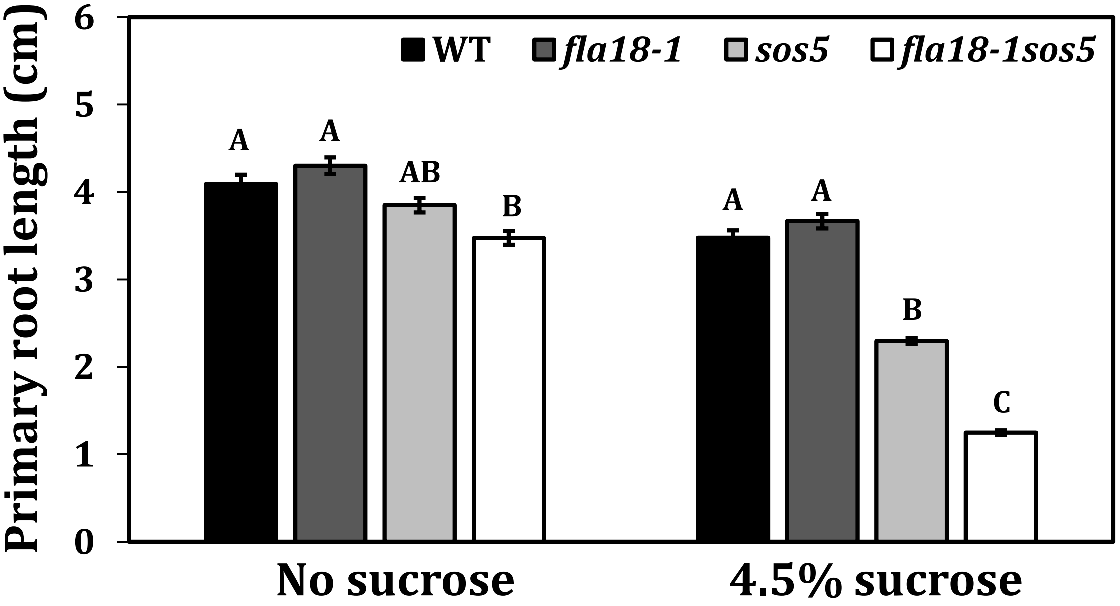

Figure S8. Primary root length of *fla18-1sos5*. Seedlings of the indicated genotypes were grown on MS medium with no sucrose for 4 d and then transfer to either permissive (sucrose-free MS) or restrictive (MS plus 4.5% sucrose) conditions. Primary root length was measured 5 d after the transfer. The results were analyzed using Tukey's HSD (Honestly significant difference) test ($p<0.05$). n=10 (*fla18-1*; n=5). Error bars represent SE.

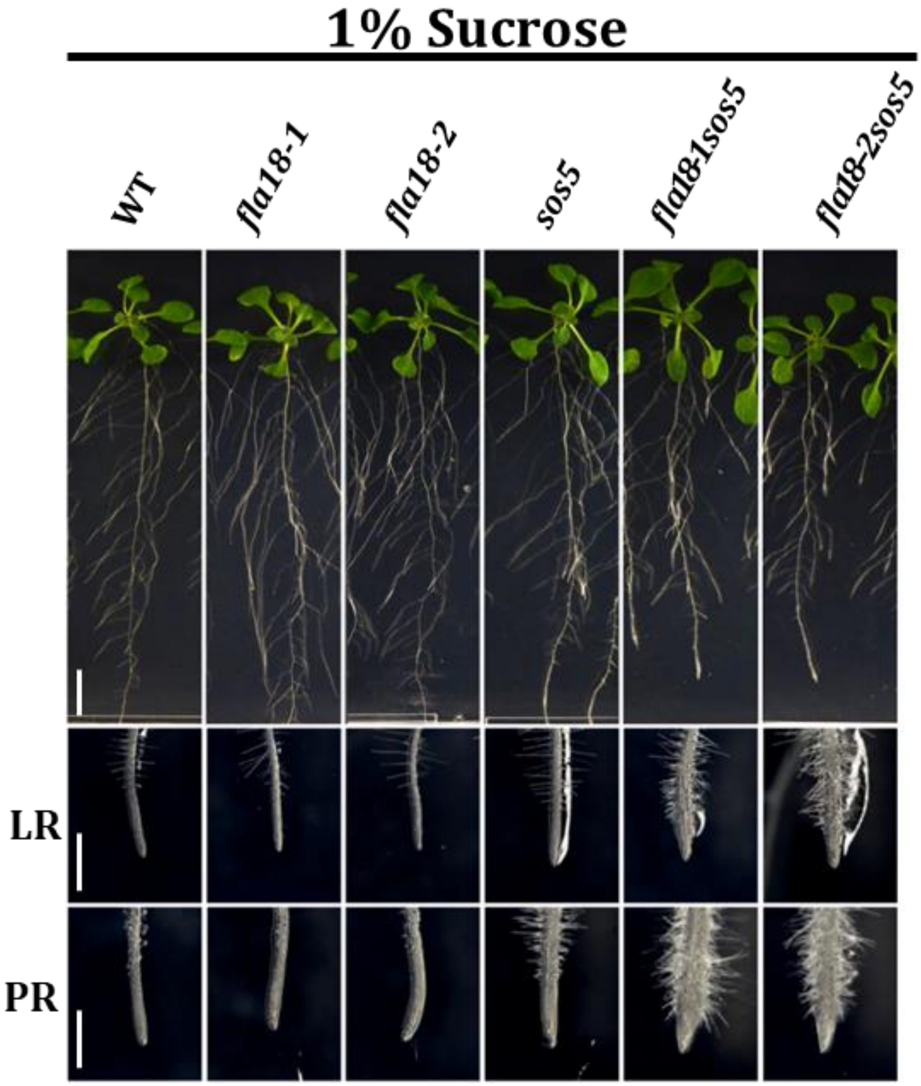

Figure S9. The *fla18sos5* double mutant phenotype on media containing 1% sucrose. Seedlings of the indicated genotypes were grown on MS medium with no sucrose for 4 d and then transferred to media containing 1% sucrose for additional 7 d. Whole seedlings (upper panel) lateral root tips (LR; middle panel) and primary root tips (PR; lower panel) were documented. Scale bars: 1 cm (upper panel), 1 mm (middle and lower panel).

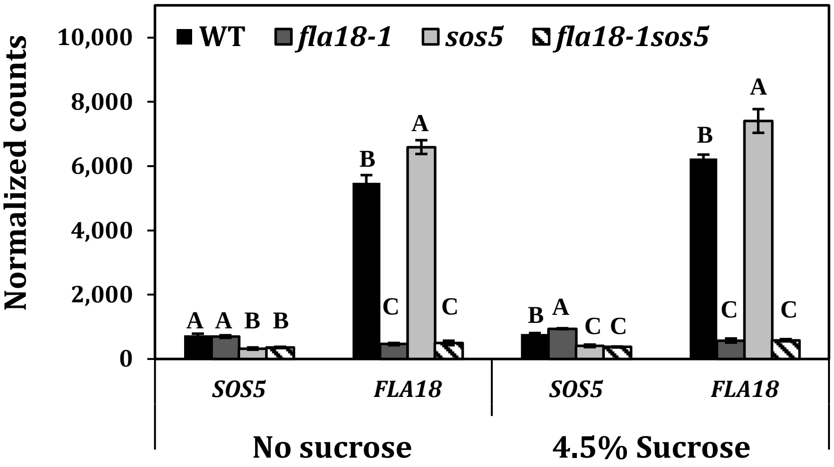

Figure S10. *FLA18* and *SOS5* gene expression in *fla18-1, sos5* and the double mutant *fla18-1sos5*. Seedlings of indicated genotypes were germinated on MS medium containing 1% sucrose, for 4 d, and then transferred to MS medium containing either no sucrose or 4.5% sucrose for 6 h. Gene expression of the indicated genes was examined in the PR using nCounter NanoString technology, in 4 biological replicates. Expression level is indicated by normalized counts, as calculated by the nSolver platform using *GAPC, UBQ10, EF1a, F-BOX* and *AP2* as reference genes. The results were analyzed using JMPpro15 for statistical analysis using Tukey's HSD (honestly significant difference) test ($p<0.05$). n=4 {except WT (No sucrose) and *sos5* (4.5% sucrose); n=3}. Error bars represent SE.

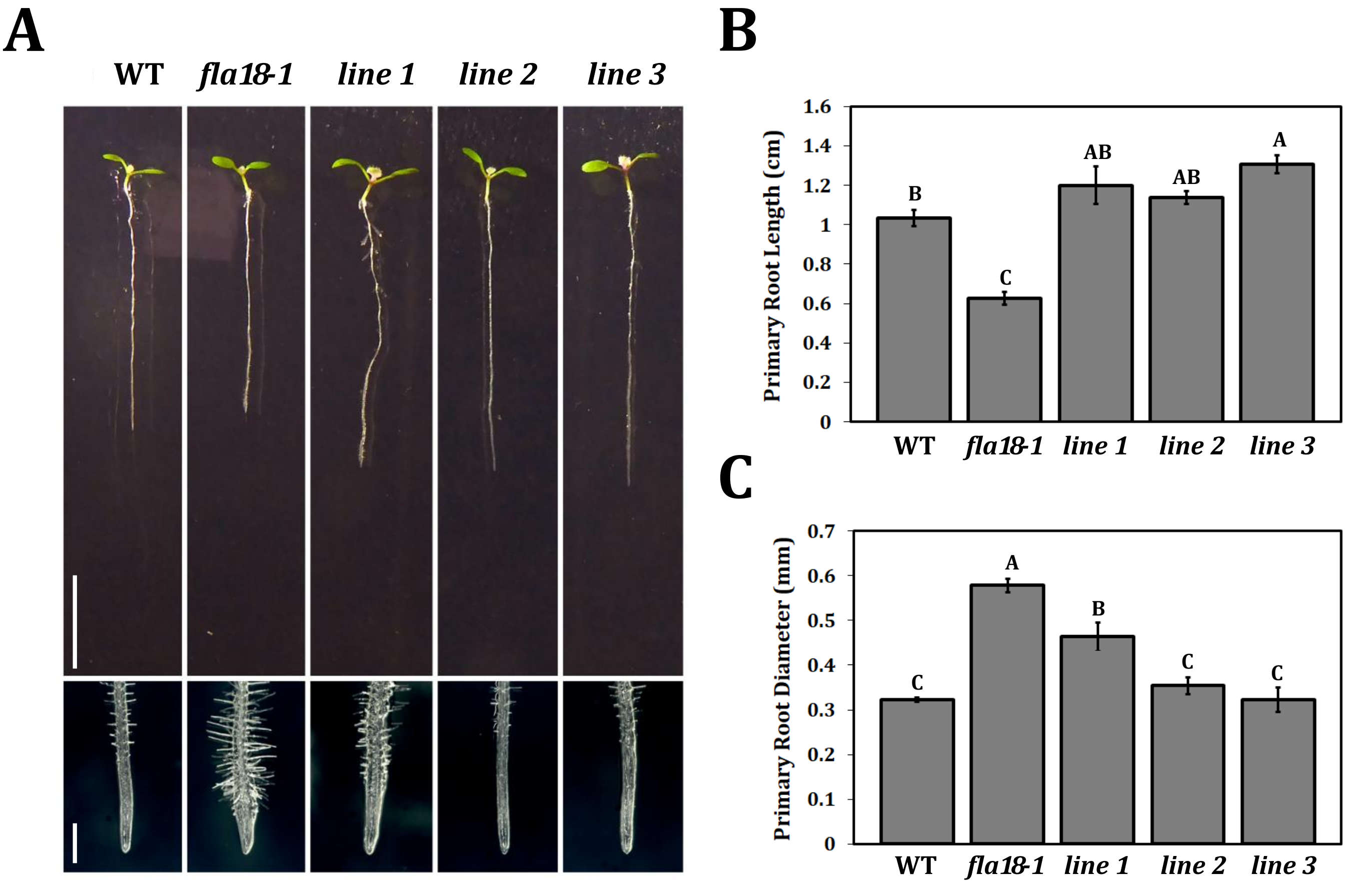

Figure S11. Expression of *FLA18* suppresses the *fla18-1* primary root hyper-sensitivity to the ABA-synthesis inhibitor, Fluridon. (A) Seedlings of the indicated genotypes and three independent *fla18-1* transgenic lines, expressing the *FLA18* CDS driven by the 35S promoter, were grown on MS medium with 1% sucrose for 5 d and then transferred to medium containing 4.5% sucrose and an addition of 5uM Fluridon for additional 2 d. Measurement of primary root length (B) and diameter (C) were conducted 48 h after the seedlings were transferred to the Fluridon containing media. The results were analyzed using JMPpro13 for statistical analysis and run through Tukey's HSD (Honestly significant difference) test (B; *p<0.001*) (C; *p<0.05).* n=10 (*line 2*; n=5). Error bars represent SE. Scale bars: 1 cm (A; upper panel), 1 mm (A; lower panel).

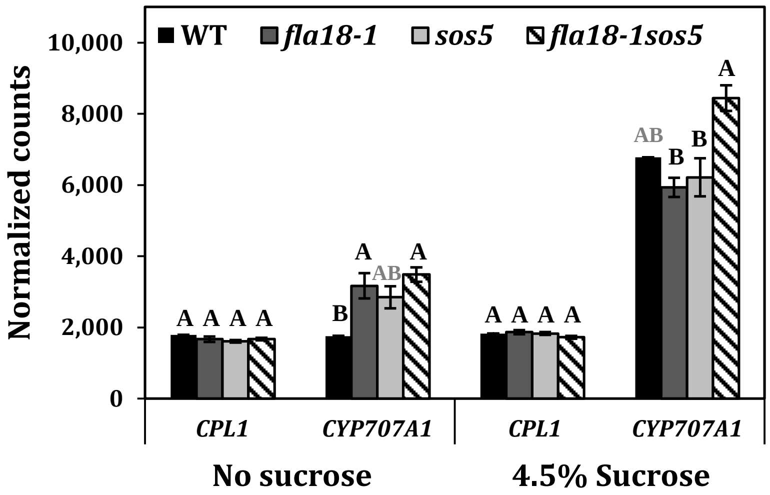

Figure S12. Expression of genes involved in ABA signaling and metabolism in *fla*-mutant background. Expression levels of *CPL1* (negative regulator of ABA induced stress-response genes) and *CYP707A1* (involved in ABA catabolism) were examined in the PR of the indicated genotypes. Seedlings were germinated on MS medium with 1% sucrose, for 4 d, and then transferred to MS medium containing either no sucrose or 4.5% sucrose for 6 h. Gene expression was measured using nCounter NanoString technology, in 4 biological replicates. Expression level is indicated by normalized counts, as calculated by the nSolver platform using *GAPC, UBQ10, EF1a, F-BOX* and *AP2* as reference genes. The results were analyzed using JMPpro15 for statistical analysis and analyzed using Tukey's HSD (Honestly significant difference) test ($p<0.05$). n=4 {except WT (No sucrose) and *sos5* (4.5% sucrose); n=3}. Error bars represent SE.

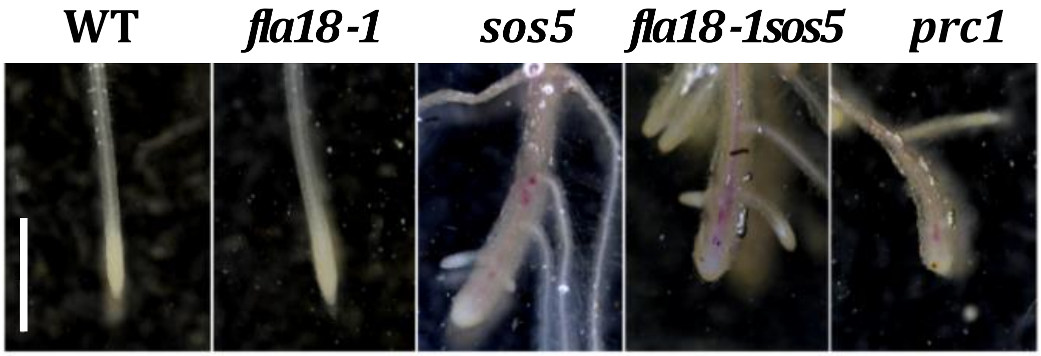

Figure S13. Histochemical Staining of the *fla18-1sos5* double mutant with phloroglucinol. Seedlings of the indicated genotypes were grown on MS medium with no sucrose for 4 d and then transferred to restrictive conditions (MS plus 4.5% sucrose) for 7 d. Phloroglucinol staining for ectopic lignin deposition in primary roots of the indicated genotypes was conducted. Scale bar: 1 mm .

Table S1

The genes used as 'baits' for the tissue-specific co-expression analysis.

ID	Name	Annotation
Root		
**AT3G16920**	*CTL2*	Chitinase-like protein 2
**AT2G22125**	*CSI1*	Cellulose synthase-interacting protein 1
**AT5G60920**	*COB*	COBRA
**AT5G63840**	*RSW3*	Alpha-subunit of a glucosidase II enzyme
**AT5G64740**	*CESA6*	Cellulose synthase A 6
**AT3G08550**	*KOB1*	Elongation defective 1 protein/ ELD1 protein
**AT5G49720**	*TSD1*	Glycosyl hydrolase 9A1
**AT1G31420**	*FEI1*	Leucine-rich repeat protein kinase family protein
**AT1G77460**	*CSI3*	Armadillo/beta-catenin-like repeat; C2 calcium/lipid-binding domain (CaLB) protein
**AT5G05170**	*CESA3*	Cellulose synthase A 3
**AT2G35620**	*FEI2*	Leucine-rich repeat protein kinase family protein
**AT1G05850**	*CTL1*	Chitinase family protein
**AT4G32410**	*CESA1*	Cellulose synthase A 1

Table S2

Primers used in this study.

Genotyping	
**Primer Name**	**Sequence**
**FLA18-1_SALK_086944 (LP)**	5’ TTATGACCATTGGAATTGTTGC
**FLA18-1_SALK_086944 (RP)**	5’ GATTAAAAGCCGTTCGATTCC
**FLA18-2_SALK-039619 (LP)**	5’ AAAAAGATTAAGCCGACGCTG
**FLA18-2_SALK_039619 (RP)**	5’ CGTTGAAATGTTTGTGTGCAC
**SOS5-2 (RP)**	5' GAAACTGGGAATAACCTTCGG
**SOS5-2 (LP)**	5' AGCTTCTCGAGACCAAACCTC
**INSERT LBb1.3**	5’ TTTTGCCGATTTCGGAAC
**Cloning**	
**Primer Name**	**Sequence**
**FLA18_promoter-Forward**	5’ CACCCTATGAGGACCACAACAATCAATCAG
**FLA18_promoter-Reverse**	5’ GGTGCTCTGTAAGATTGAGATAATCTGTAG
**FLA18_CDS-Forward**	5' CACCATGGATCGTTGTATCTATGGTTGC
**FLA18_CDS-Reverse**	5' TCAGCATCTGCTTAGATATGAGTCC
**RT-PCR**	
**Primer Name**	**Sequence**
**RT-PCR-FLA18-Forward**	5’ CCCATTTTTATCATCTAGTACTCGC
**RT-PCR-FLA18-Reverse**	5’ CGCAACAATTCCAATGGTCATA
**TUBULIN CDS- Forward**	5’ AAACTCACTACCCCCAGCTTTG
**TUBULIN CDS- Reverse**	5’ GAGAGGAGCAAAACCAACCA

Table S3

List of probes used in the nCounter NanoString analysis for gene expression.

Name	Accession	Target Sequence
**EF1a**	AK318784.1	TACCTCCCAGGCTGATTGTGCTGTTCTTATCATTGACTCCACCACTGGAGGTTTTGAGGCTGGTATCTCTAAGGATGGTCAGACCCGTGAGCACGCTCTT
**UBC10**	NM_180850.3	TAATACGAGAAGAAAAAGGCGAAAACCTCGCCAATCCGATTACGCGAAAAATCAAAGGTTTTTGGATATGGCGTCGAAGCGGATCTTGAAGGAATTGAAG
**F-box**	NM_121575.5	AGTTGGAGGACTCTACCGAGTATGCACTATAACCAACAAAGGCAATTGATTATGGTCGTGGATCGCTCAGACAAATCGTTCAAAGTCATAGCCACAAGTG
**AP2**	NM_203166.2	GTCTTGAGAAGGAATCAGAAATGAAATCTCGCCCAGCTAAGAGTGGTAAAACCATTGAGCTTGATGATGTCACATTTCACCAGTGTGTGAACCTGACCAG
**CYP707A1**	NM_202845.1	ATTTGATCCATCAAGATTCGAGGTGGCTCCAAAACCCAATACGTTCATGCCATTTGGCAATGGAACCCACTCGTGTCCTGGAAATGAATTAGCCAAGCTT
**FLA18**	NM_112002.3	GGCATGACCAAGTGCTGCATTTGAGTAAACTCAAAGGAACAAATGGAAAGAGATTGGTGAATTCCGCTGTAATTACCCGACCCGATGATTTAACCCGACC
**GAPC**	NM_111283.4	CTTATGACTACAGTCCACTCAATCACTGCTACTCAGAAGACTGTTGATGGGCCTTCAATGAAGGACTGGAGAGGTGGAAGAGCTGCTTCATTCAACATTA
**RD29B**	NM_001036984.1	GAAGATTTTCCGACAAGAGGTGATGTGAAAGTAGAGAGTGGATTGGGAAGAGACTTACCGACGGGAACTCATGATCAGTTCTCACCAGAACTATCTCGTC
**FLA4**	NM_114522.4	GGAATCAATCTCACTCAGATACTAATCAACGGACACAACTTCAACGTCGCTCTATCCCTCCTCGTCGCTTCCGGTGTCATAACAGAATTCGAAAACGACG
